# Construction and Validation of Novel Nomograms for Predicting Prognosis of Pancreatic Ductal Adenocarcinoma After Surgery According to Different Primary Cancer Locations

**DOI:** 10.3389/fonc.2021.646082

**Published:** 2021-04-23

**Authors:** Ge Li, Cheng-Yu Liao, Jiang-Zhi Chen, Long Huang, Can Yang, Yi-Feng Tian, Yi-Ting Wang, Qiang Du, Qian Zhan, Yan-Ling Chen, Shi Chen

**Affiliations:** ^1^ Department of Hepatobiliary Surgery and Fujian Institute of Hepatobiliary Surgery, Fujian Medical University Union Hospital, Fuzhou, China; ^2^ Key Laboratory of The Ministry of Education for Gastrointestinal Cancer, Fujian Medical University, Fuzhou, China; ^3^ Shengli Clinical Medical College of Fujian Medical University, Fujian Medical University, Fujian Provincial Hospital, Fuzhou, China; ^4^ Fujian Provincial Key Laboratory on Hematology, Fujian Institute of Hematology, Fujian Medical University Union Hospital, Fuzhou, China; ^5^ Pancreatic Disease Center, Department of General Surgery, Ruijin Hospital, Research Institute of Pancreatic Diseases, Shanghai Jiao Tong University School of Medicine, Shanghai, China

**Keywords:** pancreatic ductal adenocarcinoma, nomogram, cancer-specific survival (CSS), decision curve analysis, AJCC 8

## Abstract

**Background/Aims:**

Pancreatic ductal adenocarcinoma (PDAC) can occur in different parts of the pancreas. This study aimed to identify clinicopathological characteristics independently correlated with the prognosis of PDAC of the pancreatic head/uncinate (PHC) or body-tail (PBTC), and to develop novel nomograms for predicting cancer-specific survival (CSS) according to different primary cancer locations.

**Methods:**

1160 PDAC patients were retrospectively enrolled and assigned to training and test sets with each set divided into PHC and PBTC groups. Comparative analysis of clinicopathologic characteristics, survival analysis, and multivariate analysis were performed. Independent factors were identified and used for constructing nomograms. The performance of the nomograms was validated in the test set.

**Results:**

Primary tumor location was an independent risk factor for prognosis of PDAC after surgery. Specially, gender, fasting blood glucose, and preoperative cancer antigen 19-9 were significantly associated with prognosis of PHC, whereas age, body mass index, and lymph nodes were significantly correlated with the prognosis of PBTC. A significant difference in prognosis was found between PHC and PBTC in stage Ia and stage III. Three nomograms were established for predicting the prognosis for PDAC, PHC, and PBTC. Notably, these nomograms were calibrated modestly (c-indexes of 0.690 for PDAC, 0.669 for PHC, and 0.704 for PBTC), presented better accuracy and reliability than the 8^th^ AJCC staging system, and achieved clinical validity.

**Conclusions:**

PHC and PBTC share the differential clinical-pathological characteristics and survival. The nomograms show good performance for predicting prognosis in PHC and PBTC. Therefore, these nomograms hold potential as novel approaches for predicting survival of PHC and PBTC patients after surgery.

## Introduction

Pancreatic ductal adenocarcinoma (PDAC), a predominate type of pancreatic cancer (PC), is among the leading causes of cancer-related death, accounting for approximately 260,000 deaths worldwide annually (1). It has been recognized in recent decades that PDAC has an extremely poor prognosis with a 5-year survival rate of less than 10%. For PDAC patients eligible for surgical treatment, curative-intent surgical resection followed by adjuvant chemotherapy is considered the only curative treatment option (2). Although substantial progress has been made in the diagnosis and treatment of PDAC, early relapse after pancreatectomy commonly occurs in PDAC patients. Thus, an accurate prognostic method is urgently required for the precise stratification of patients to guide appropriate clinical management and follow-up plans.

Currently, the stratification of patients mainly relies on the American Joint Committee on Cancer (AJCC) staging system, in which tumor size and the histological characteristics are considered as the major factors for evaluation. However, many significant factors, such as cancer antigen 19-9 (CA19-9) level, tumor differentiation, histological grade, and genomic analysis, have been proposed to be potential determinants of survival but have not been included in the AJCC staging system. Moreover, PDAC can occur in various parts of the pancreas (head, body or tail) of the pancreas, and the risk factors influencing the prognosis of PDAC according to different primary locations have not been thoroughly investigated.

A number of previous studies have indicated that tumor location is closely related to the prognosis of PC, with primary tumor location at the body/tail of the pancreas (PBTC) tending to have a poorer prognosis compared with that at the head of the pancreas (3–6). Additionally, resected PBTC has shown more aggressive tumor biology than PDAC at the head of the pancreas. On the contrary, some previous studies demonstrated that PBTC had a better outcome than PDAC at the head of the pancreas for patients at the early stage (7), and Winer et al. (8) found that patients with pancreatic head cancer had worse overall survival (OS) than patients with PC at either the body or tail locations for all stages when analyzing the National Cancer Database. Nevertheless, van Erning et al. (9) indicated that OS for PDAC at different tumor locations does not differ significantly according to the database in the Netherlands. Thus, the findings regarding the prognosis of PDAC at different primary tumor locations remain inconsistent and even conflicting. Use of the TNM staging system for stratifying PDAC patients in order to determine the precise prognosis is questionable (10). Until now, research of the survival difference for PDAC at different locations after curative-intent surgical resection has been rare.

In the present study, we aimed to identify clinicopathological characteristics independently correlated with the prognosis of PDAC at the pancreatic head/uncinate (PHC) versus the PBTC and to develop and validate novel nomograms for predicting the cancer-specific survival (CSS) of PDAC cases according to different primary cancer locations after curative-intent resection. The findings of this study may provide a novel prognostic tool for managing PDAC cases with different primary tumor locations.

## Methods

### Patients and Study Design

A total of 1160 PDAC patients who underwent curative-intent pancreatic resection at multiple centers, including Fujian Medical University Union Hospital, Fujian Provincial Hospital, and Shanghai Ruijin Hospital, spanning the period between January 2014 and March 2017 were retrospectively enrolled in this study. PDAC was histopathologically diagnosed and confirmed. Of 1160 enrolled patients, 813 enrolled from Fujian Medical University Union Hospital and Fujian Provincial Hospital were assigned to the training group, while 347 from Shanghai Ruijin Hospital were assigned to the test group for external validation. During enrollment, the following inclusion criteria were used: (1) histologically confirmed PDAC; (2) no prior receipt of other curative treatment including radiotherapy, immunogene and target therapy; (3) curatively resectable PDAC as preoperatively assessed by imaging, even with peripancreatic invasion or artery (hepatic, superior mesenteric and celiac artery) or vein (portal or superior and inferior mesenteric vein) that could be completely resected and constructed; (4) negative for intraoperative frozen section analysis; (5) only the single metastatic lesion in liver for patients with stage IV disease after 8-12 times paclitaxel-albumin or gemcitabine chemotherapy. PDAC patients with the following conditions were excluded from this study: (1) absent or incomplete information for important clinical characteristics needed for this study; (2) unresected tumors, based on bypass surgery, exploratory operation, or microscopic residual tumor in the resection margin; (3) death within 30 days after the surgery; and (4) causes of death other than PDAC and its complications.

This study was approved by the ethics committees of the institutional review boards of the participating hospitals, Fujian Medical University Union Hospital, Fujian Provincial Hospital, and Shanghai Ruijin Hospital. The need for written consent was waived due to the retrospective nature of this study.

### Follow-Up

The patients were followed up by the operating surgeons at 1 month after surgery and every 3 months thereafter. The last follow-up was conducted in March 2020.

### Statistical Analysis

Categorical variables were compared between groups using the chi square test. Continuous variables were analyzed using the independent-samples T test. To assess an association between various prognostic predictors and CSS, univariate and multivariate analyses were conducted using the Cox regression model, and hazard ratios (HRs) and 95% confidence intervals (CIs) were calculated. Goodness of fit was maximized using the log-likelihood, while information loss was minimized with the Akaike information criterion (AIC) (11). Based on the AIC of the Cox proportional hazards model, variables were selected in a backward stepwise manner. Nomograms were constructed on the basis of the independent variables identified by the multivariate analysis in the training cohort.

Nomogram performance was assessed using the Harrell’s concordance index (c-index). The maximum c-index value of 1.0 represents a perfect discrimination. whereas 0.5 indicates no discriminative capacity. Calibration was made to graphically evaluate the performance of the model by comparing the means of predicted survival with those of actual survival. To reduce potential bias, 200-sample bootstrap validation was performed for internal validation. The values of c-indexes were compared using the compare C package (12). The precision of the 1-, 2-, and 3-year survival rates predicted by the nomograms was evaluated with time-dependent receiver operating characteristic (ROC) curve analysis using the time ROC package (13).

The ranges of threshold probabilities were finalized by decision curve analysis (DCA) (14) to assess the clinical validity of the nomograms. The Kaplan–Meier survival curve was utilized for comparing the nomograms with the latest edition of the 8^th^ AJCC staging system (revised in 2018) by risk classification and stratification (15). For risk stratification, the accumulated nomogram scores were ranked by deciles to develop 10 risk groups, which composed the new nomo stages. Accordingly, each 8^th^AJCC substage was divided by nomo stages to derive three prognostic strata: low-, median-, and high-risk.

## Results

### Baseline Demographic and Clinical Characteristics of PDAC Patients

A total of 1160 patients with PDAC were enrolled from three participating hospitals, of which 813 (467 PHC cases and 346 PBTC cases) recruited from Fujian Medical University Union Hospital and Fujian Provincial Hospital were assigned to the training set. The baseline demographic and clinical characteristics of the PHC and PBTC patients are summarized in **Table 1**. The age and gender of patients were comparable between the training and test cohorts in both PHC and PBTC groups (**Suppl. Tables 1**, **2**). It was noted that a majority of PDAC patients (PHC and PBTC groups) were men, with a greater male predominance in the PHC group (64.67% males and 35.33% females) than in the PBTC group (p=0.001). Comparison of clinical features revealed that patients in the PHC group showed a higher proportion of symptoms (e.g., jaundice, abdominal pain; p<0.001), thus leading to more timely medical intervention for PHC patients than PBTC patients. It was of note that PHC patients presented the earlier T and AJCC stage and less hepatic metastasis compared with PBTC patients (p<0.001). In addition, PHC patients exhibited a higher number of harvested lymph nodes confirmed by postoperative pathology, which described as lymph node count (LNC) afterward, than PBTC patients (11.27 and 4.86 for PHC and PBTC, respectively). The number of lymph nodes dissected during operation might be enough, but many of them were confirmed as adipose tissue by postoperative pathology, which could be the reason for that LNC in this study was lower than the number of 8^th^ AJCC or ISGPS (International Study Group of Pancreatic Surgery) recommend. Lymph node metastasis occurred more frequently in the PHC group compared with the PBTC group (261 vs 219 in N0, 161 vs 108 in N1, 45 vs 19 in N2, p<0.001). Notably, the PBTC group had better tumor differentiation, less intraoperative blood loss, and higher values for lymph node ratio (LNR), albumin, carcino-embryonic antigen (CEA) and cancer antigen 125 (CA125) compared with the PHC group. The demographic and clinical characteristics were comparable among the patients in the training and test groups.

**Table 1:** Baseline demographic and clinical characteristics of the patients.

Variables	PHC		PBTC		p
	n=467		n=346		
Gender, n, (%)					0.001
Female	165	(35.33)	166	(47.98)	
Male	302	(64.67)	180	(52.02)	
Age (years), median, IQR	62.00	56.00, 69.00	63.00	57.00, 70.00	0.074
BMI (kg/m^2^), mean, SD	22.88	3.44	22.83	3.20	0.831
Symptoms, (%)					<0.001
Yes (including abdominal pain/gastrointestinal symptoms)	350	(74.95)	218	(63.01)	
No	117	(25.05)	128	(36.99)	
TBIL (umol/L), mean, SD	97.24	94.03	15.28	5.90	<0.001
ALB (g/L), mean, SD	36.58	5.25	39.42	4.32	<0.001
Fasting blood glucose (mmol/L), mean, SD	6.42	2.24	6.85	4.99	0.104
CA125, (%)					0.002
Normal	396	(84.80)	264	(76.30)	
Elevated	71	(15.20)	82	(23.70)	
CA19-9, (%)					0.492
Normal	108	(23.13)	73	(21.10)	
Elevated	359	(76.87)	273	(78.90)	
CEA, (%)					<0.001
Normal	351	(75.16)	216	(62.43)	
Elevated	116	(24.84)	130	(37.57)	
Smoking history, (%)					0.549
Yes	122	(26.12)	84	(24.28)	
No	345	(73.88)	262	(75.72)	
Drinking history, (%)					0.638
Yes	87	(18.63)	69	(19.94)	
No	380	(81.37)	277	(80.06)	
Histology, n, (%)					<0.001
Well differentiated	18	(3.85)	44	(12.72)	
Moderately differentiated	232	(49.68)	143	(41.33)	
Poorly differentiated	217	(46.47)	159	(45.95)	
Intraoperative blood loss (ml), mean, SD	613.19	488.26	505.00	487.05	0.002
Tumor size (cm), mean, SD	3.23	1.30	4.44	1.88	<0.001
Perineuronal invasion n, (%)					0.332
Yes	295	(63.17)	207	(59.83)	
No	172	(36.83)	139	(40.17)	
pT stage, n, (%)					<0.001
pT1	102	(21.84)	25	(7.23)	
pT2	281	(60.17)	163	(47.11)	
pT3	77	(16.49)	120	(34.68)	
pT4	7	(1.50)	38	(10.98)	
pN stage, n, (%)					0.033
N0	261	(55.89)	219	(63.29)	
N1	161	(34.48)	108	(31.21)	
N2	45	(9.64)	19	(5.49)	
LNC, mean, SD	11.27	8.51	4.86	5.17	<0.001
LNR, mean, SD	0.11	0.19	0.18	0.32	<0.001
LNM, mean SD	1.16	1.96	0.92	2.29	0.109
Metastasis, n, (%)					<0.001
M0	442	(94.65)	293	(84.68)	
M1	25	(5.35)	53	(15.32)	
8^th^ AJCC stage, n, (%)					<0.001
Ia	64	(13.70)	18	(5.20)	
Ib	150	(32.12)	96	(27.75)	
IIa	35	(7.49)	64	(18.50)	
IIb	147	(31.48)	72	(20.81)	
III	46	(9.85)	43	(12.43)	
IV	25	(5.35)	53	(15.32)	
Neoadjuvant chemotherapy, n, (%)					0.722
Yes	131	(28.05)	101	(29.19)	
No	336	(71.95)	245	(70.81)	
1-year cumulative survival	0.665		0.589		<0.001
2-year cumulative survival	0.451		0.312		
3-year cumulative survival	0.322		0.186		

PHC, pancreatic head/uncinate ductal adenocarcinoma; PBTC, pancreatic body/tail ductal adenocarcinoma; IQR, interquartile range; SD, standard deviation; BMI, body mass index; TBIL, total bilirubin; ALB, albumin; LNC, lymph node count; LNM, lymph node metastasis; LNR, lymph node ratio; CA125, cancer antigen 125; CA19-9, cancer antigen 19-9; CEA, carcino-embryonic antigen.

The median survival was 20 months (range, 1–74 months) and the 1-, 2-, and 3-year survival rates were 66.5%, 45.1%, and 32.2% in the PHC group, respectively. For patients with PBTC, the median survival was 14 months (range, 1–74 months), and the 1-, 2-, and 3-year survival rates were 58.9%, 31.2%, and 18.6%, respectively. Notably, PBTC patients had a significantly worse CSS compared with PHC patients (p<0.001).

### Survival Analysis for Patients With PHC and PBTC

**Figure 1:**
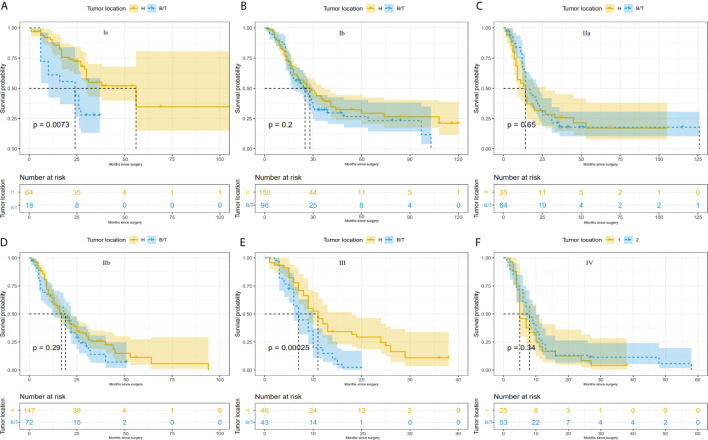
Comparison of survival differences between PHC and PBTC according to the 8^th^AJCC system. Comparison between PHC and PBTC in **(A)** stage Ia; **(B)** stage Ib; **(C)** stage IIa; **(D)** stage IIb; **(E)** stage III; and **(F)** stage IV. Significant differences were observed in Ia and III (p=0.0073 and 0.00025, respectively).

Survival rates were compared between the PHC and PBTC groups according to the AJCC stages (**Figure 1**). As a result, significant differences in survival were found in stage Ia and stage III, and patients in the PHC group had poorer clinical outcomes than those in the PBTC group (p=0.007 and <0.001 in stage Ia and stage III, respectively). The differences in clinical characteristics between the PHC and PBTC groups in stage Ia and stage III are summarized in **Suppl. Tables 3**, **4**. In stage Ia, patients with PBTC showed significantly worse tumor differentiation (p=0.012) and a lower LNC (p=0.001) compared to patients with PHC. In stage III, patients in the PBTC group had a significantly larger tumor size (p=0.004) and later T stage (p<0.001) than patients in the PHC group, while the PHC group showed significantly more lymph nodes metastasis (p=0.015) and later N stage (p<0.001), with a higher LNC (p<0.001) than the PBTC group.

### Identification of Independent Prognostic Factors for PHC and PBTC After Curative-Intent Surgical Resection

Univariate and multivariate analyses were conducted to identify prognostic factors that correlate with different primary cancer locations of PDAC, including PHC and PBTC, and detailed results are listed in **Tables 2**–**4**. In all enrolled PDAC patients with any primary cancer location, tumor location, gender, age, BMI, histological grade, symptoms, fasting blood glucose, tumor size, perineuronal invasion, T category, N category, hepatic metastasis, LNR, lymph node metastasis (LNM), and preoperative levels of CA19-9, CA125, and CEA were identified to be significantly associated with survival (**Table 2**). Further, multivariate analysis revealed that primary tumor location was an independent prognostic factor (PBTC: hazard ratio [HR] 1.443, 95% CI, 1.225–1.699, p<0.0001). In addition, gender, BMI, histological grade, symptoms, fasting blood glucose, tumor size, perineuronal invasion, M category, LNR, LNM, and preoperative CA19-9 and CEA levels were also independent prognostic factors for PDAC (**Table 2**).

**Table 2:** Univariate and multivariate Cox regression analyses of prognostic factors in PDAC patients with curative-intent surgical resection.

Variables	Univariate analysis			Multivariate analysis			
	HR	95%CI	p	HR	95%CI	p	
Tumor location							
Head	ref			ref			
Body/Tail	1.443	1.225-1.699	<0.0001	1.273	1.057-1.535	0.011	
Gender							
Male	ref			ref			
Female	0.842	0.712-0.996	0.044	0.776	0.653-0.924	0.004	
Age	1.009	1.000-1.018	0.041	1.006	0.997-1.016	0.173	
BMI	0.975	0.951-0.999	0.038	0.958	0.935-0.983	0.001	
Symptoms							
No	ref			ref			
Yes	1.283	1.069-1.539	0.007	1.231	1.020-1.488	0.030	
TBIL	1.000	0.999-1.001	0.936	–			
ALB	0.992	0.976-1.008	0.329	–			
Fasting blood glucose	1.059	1.036-1.082	<0.0001	1.038	1.013-1.063	0.003	
CA125							
Normal	ref			ref			
Elevated	1.698	1.392-2.073	<0.0001	1.213	0.968-1.520	0.093	
CA19-9							
Normal	ref			ref			
Elevated	1.404	1.145-1.720	0.001	1.324	1.077-1.629	0.008	
CEA							
Normal	ref			ref			
Elevated	1.210	1.016-1.441	0.03	1.110	1.013-1.209	0.026	
Smoking history							
No	ref			–			
Yes	1.092	0.908-1.313	0.35	–			
Drinking history							
No				–			
Yes	1.060	0.863-1.302	0.58	–			
Histology							
Poorly differentiated	ref			ref		
Moderately differentiated	0.635	0.534-0.754	<0.0001	0.722	0.605-0.861	0.0002
Well differentiated	0.656	0.478-0.901	0.009	0.656	0.472-0.913	0.012
Intraoperative blood loss	1.000	0.999-1.000	0.121	–			
Tumor size	1.179	1.127-1.233	<0.0001	1.123	1.070-1.184	<0.0001	
Perineuronal invasion							
No	ref			ref			
Yes	1.572	1.322-1.869	<0.0001	1.417	1.184-1.697	0.0001	
pT stage							
pT1	ref			–			
pT2	1.357	1.050-1.753	0.019	–			
pT3	2.017	1.528-2.661	<0.0001	–			
pT4	9.600	6.439-14.312	<0.0001	–			
pN stage							
N0	ref			–			
N1	1.594	1.334-1.905	<0.0001	–			
N2	2.444	1.824-3.274	<0.0001	–			
LNC	0.995	0.984-1.006	0.358	–			
LNR	2.067	1.604-2.664	<0.0001	1.527	1.093-2.132	0.012	
LNM	1.088	1.059-1.118	<0.0001	1.055	1.020-1.092	0.002	
Metastasis							
M0	ref			ref			
M1	2.940	2.296-3.764	<0.0001	2.115	1.572-2.847	<0.0001	
8^th^ AJCC stage							
Ia	ref			–			
Ib	1.342	0.946-1.902	0.099	–			
IIa	2.076	1.416-3.042	0.0001	–			
IIb	2.167	1.530-3.069	<0.0001	–			
III	4.853	3.297-7.145	<0.0001	–			
IV	5.456	3.702-8.043	<0.0001	–			
Neoadjuvant chemotherapy							
No	ref			ref			
Yes	0.832	0.691-1.001	0.05	0.787	0.651-0.952	0.013	

PDAC, pancreatic ductal adenocarcinoma; BMI, body mass index; TBIL, total bilirubin; ALB, albumin; LNC, lymph node count; LNM, lymph node metastasis; LNR, lymph node ratio; CA125, cancer antigen 125; CA19-9, cancer antigen 19-9; CEA, carcino-embryonic antigen.

**Table 3:** Univariate Cox regression analysis of prognostic factors in PHC and PBTC patients with curative-intent surgical resection.

Variables	Univariate analysis for PHC			Univariate analysis for PBTC		
	HR	95%CI	p	HR	95%CI	p
Gender						
Male	ref			ref		
Female	0.718	0.564-0.916	0.008	0.907	0.714-1.154	0.428
Age	1.002	0.990-1.015	0.699	1.016	1.003-1.029	<0.0001
BMI	0.990	0.959-1.022	0.522	0.948	0.912-0.985	0.006
Symptoms						
No	ref			ref		
Yes	1.229	0.944-1.601	0.126	1.528	1.185-1.971	0.001
TBIL	1.002	1.000-1.003	0.008	1.006	0.986-1.027	0.569
ALB	0.983	0.962-1.004	0.113	0.972	0.943-1.002	0.071
Fasting blood glucose	1.066	1.019-1.115	0.005	1.054	1.025-1.083	0.0002
CA125						
Normal	ref			ref		
Elevated	1.465	1.092-1.967	0.011	1.840	1.399-2.421	<0.0001
CA19-9						
Normal	ref			ref		
Elevated	1.509	1.140-1.997	0.004	1.276	0.948-1.717	0.109
CEA						
Normal	ref			ref		
Elevated	1.014	0.786-1.311	0.920	1.355	1.063-1.728	0.014
Smoking history						
No	ref			ref		
Yes	0.983	0.764-1.265	0.896	1.337	1.018-1.756	0.037
Drinking history						
No	ref			ref		
Yes	1.058	0.796-1.406	0.700	1.051	0.779-1.417	0.744
Histology						
Poorly differentiated	ref			ref		
Moderately differentiated	0.691	0.372-1.210	0.185	0.564	0.435-0.734	<0.0001
Well differentiated	0.671	0.549-0.871	0.001	0.528	0.359-0.776	0.001
Intraoperative blood loss	1.000	1.000-1.000	0.006	1.000	0.999-1.000	0.927
Tumor size	1.205	1.110-1.309	<0.0001	1.131	1.064-1.202	<0.0001
Perineuronal invasion						
No	ref			ref		
Yes	1.678	1.317-2.138	<0.0001	1.493	1.164-1.916	0.001
pT stage						
pT1	ref			ref		
pT2	1.414	1.041-1.922	0.027	1.073	0.667-1.726	0.772
pT3	2.021	1.401-2.915	0.0001	1.569	0.968-2.543	0.067
pT4	14.900	6.607-33.604	<0.0001	7.252	4.036-13.029	<0.0001
pN stage						
N0	ref			ref		
N1	1.683	1.320-2.146	<0.0001	1.622	1.244-2.115	0.0003
N2	2.484	1.711-3.607	<0.0001	3.272	2.014-5.317	<0.0001
LNC	0.996	0.983-1.010	0.602	1.043	1.019-1.066	0.0003
LNR	3.418	2.148-5.438	<0.0001	1.523	1.109-2.090	0.009
LNM	1.133	1.080-1.189	<0.0001	1.070	1.035-1.107	<0.0001
Metastasis						
M0	ref			ref		
M1	4.034	2.623-6.205	<0.0001	2.296	1.685-3.127	<0.0001
8^th^ AJCC stage						
Ia	ref			ref		
Ib	1.517	0.983-2.339	0.060	0.797	0.438-1.448	0.456
IIa	2.624	1.542-4.466	0.0003	1.072	0.581-1.981	0.822
IIb	2.499	1.635-3.819	<0.0001	1.299	0.706-2.389	0.399
III	4.057	2.452-6.711	<0.0001	4.620	2.436-8.759	<0.0001
IV	8.046	4.585-14.117	<0.0001	2.794	1.513-5.158	0.001
Neoadjuvant chemotherapy						
No	ref			ref		
Yes	0.814	0.628-1.055	0.120	0.824	0.632-1.074	0.152

PHC, pancreatic head/uncinate ductal adenocarcinoma; PBTC, pancreatic body/tail ductal adenocarcinoma; BMI, body mass index; TBIL, total bilirubin; ALB, albumin; LNC, lymph node count; LNM, lymph node metastasis; LNR, lymph node ratio; CA125, cancer antigen 125; CA19-9, cancer antigen 19-9; CEA, carcino-embryonic antigen.

**Table 4:** Multivariate Cox regression analysis of prognostic factors for PHC and PBTC patients with curative-intent surgical resection.

Variables	Multivariate analysis of PHC			Variables	Multivariate analysis of PBTC		
	HR	95% CI	p		HR	95% CI	p
Gender				Age	1.018	1.004-1.032	0.013
Male	ref			BMI	0.933	0.898-0.970	0.0004
Female	0.761	0.595-0.973	0.029	Fasting blood glucose	1.031	0.999-1.064	0.052
Fasting blood glucose	1.051	1.001-1.103	0.047	Smoking history			
Intraoperative blood loss	1.000	0.999-1.000	0.143	No	ref		
Histology				Yes	1.368	1.028-1.821	0.031
Poorly differentiated	ref			Histology			
Moderately differentiated	0.963	0.500-1.685	0.782	Poorly differentiated	ref		
Well differentiated	0.775	0.608-0.988	0.04	Moderately differentiated	0.644	0.493-0.841	0.001
Tumor size	1.112	1.017-1.216	0.019	Well differentiated	0.524	0.352-0.781	0.001
Perineuronal invasion				Tumor size	1.112	1.043-1.187	0.001
No	ref			Perineuronal invasion			
Yes	1.447	1.125-1.862	0.004	No	ref		
LNR	2.281	1.167-4.457	0.015	Yes	1.492	1.148-1.939	0.002
LNM	1.062	0.989-1.140	0.096	LNR	1.588	1.113-2.266	0.011
Metastasis				LNC	1.035	1.010-1.059	0.005
M0	ref			Metastasis			
M1	2.991	1.907-4.693	<0.0001	M0	ref		
CA125				M1	2.174	1.521-3.109	<0.0001
Normal	ref			Symptoms	0.905	0.835-0.981	0.044
Elevated	1.277	0.939-1.736	0.119	No	ref		
CA19-9				Yes	1.338	1.032-1.736	0.028
Normal	ref			CEA			
Elevated	1.612	1.204-2.157	0.001	Normal	ref		
				Elevated	1.388	1.193-1.647	0.101

PHC, pancreatic head/uncinate ductal adenocarcinoma; PBTC, pancreatic body/tail ductal adenocarcinoma; BMI, body mass index; LNC, lymph node count; LNM, lymph node metastasis; LNR, lymph node ratio; CA125, cancer antigen 125; CA19-9, cancer antigen 19-9; CEA, carcino-embryonic antigen.

The PDAC at different locations (PHC and PBTC) shared common independent prognostic factors: histological grade, tumor size, LNR, perineuronal invasion, M category and symptoms (**Table 4**). Notably, gender, fasting blood glucose, and preoperative CA19-9 level were significantly associated with the prognosis of PHC only, whereas age, BMI, and LNC were significantly correlated with the prognosis of PBTC only (**Table 4**), reflecting differences in independent prognostic factors between PHC and PBTC.

### Construction and Validation of Prognostic Nomograms for PHC and PBTC

**Figure 2:**
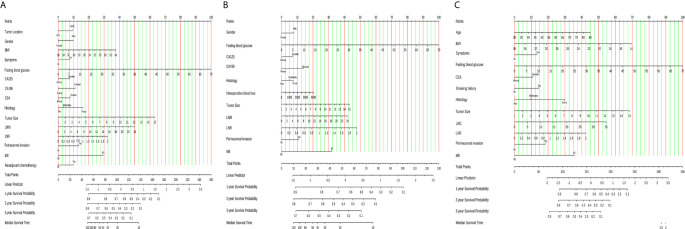
Nomograms for survival prediction in PDAC, PHC and PBTC patients after surgery. **(A)** 1-, 2-, and 3-year CSS of PDAC; **(B)** 1-, 2-, and 3-year CSS of PHC; **(C)** 1-, 2-, and 3-year CSS of PBTC. The total points for the nomograms were calculated according to the points for each covariate. A higher total number of points represented a higher possibility of unfavorable expected survival. CSS, cancer- specific survival.

The identified independent risk factors influencing the prognosis of PHC and PBTC were used to construct prognostic nomograms for PHC and PBTC. As shown in **Figure 2**, covariates were selected on the basis of the AIC and likelihood rather than statistical significance (p value) to balance model complexity and performance. Points in the nomogram could be summed to calculate the probability of individual survival. The labels and points in the nomogram are presented in detail in **Suppl. Tables 5**, **6**.

**Figure 3:**
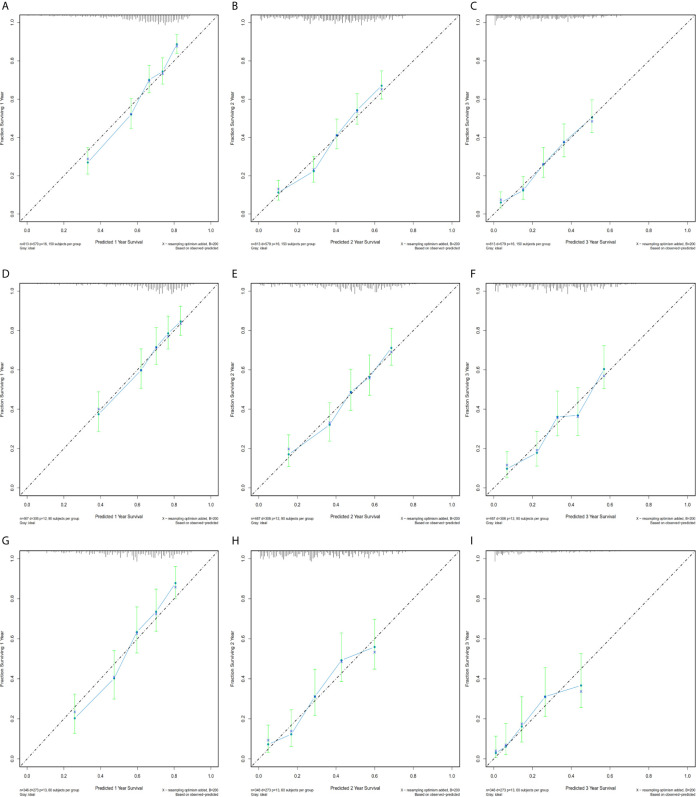
Bootstrap calibrations of the nomograms in the training cohorts. Bootstrap calibrations of the nomograms for predicting **(A)** 1-year CSS, **(B)** 2-year CSS, and **(C)** 3-year CSS in PDAC group; **(D)** 1-year CSS, **(E)** 2-year CSS, and **(F)** 3-year CSS in the PHC group; and **(G)** 1-year CSS, **(H)** 2-year CSS, and **(I)** 3-year CSS in the PBTC group. The predictions were well correlated with the actual survival probabilities.

**Figure 4:**
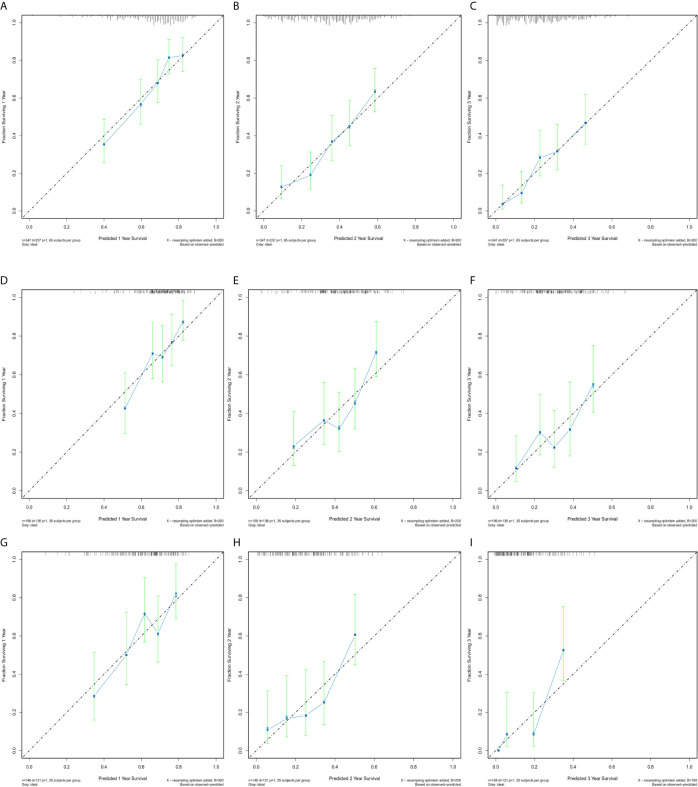
Bootstrap calibration of nomograms in the test cohorts. The nomograms were externally validated in the test cohorts by predicting **(A)** 1-year CSS, **(B)** 2-year CSS, and **(C)** 3-year CSS in the PDAC group; **(D)** 1-year CSS, **(E)** 2-year CSS, and **(F)** 3-year CSS in the PHC group; and **(G)** 1-year CSS, **(H)** 2-year CSS, and **(I)** 3-year CSS in the PBTC group. All results showed good validation.

Calibration plots were generated for the probabilities of 1-, 2-, and 3-year CSS of PDAC, PHC, and PBTC, and favorable consistency was illustrated by the survival predicted by the nomograms and the corresponding Kaplan–Meier estimates in both the training and test cohorts (**Figures 3**, **4**), indicating that the established nomograms were reliable for predicting survival.

The bootstrap-corrected c-indexes in the training cohort were 0.690 (95% CI 0.667–0.712) for PDCA, 0.669 (95% CI 0.636–0.702) for PHC, and 0.704 (95% CI 0.672–0.735) for PBTC. In the test cohort, the c-indexes were 0.669 (95% CI 0.634–0.704) for PDCA, 0.636 (95% CI 0.585–0.688) for PHC, and 0.643 (95% CI 0.588–0.699) for PBTC (**Table 5**).

**Table 5:** Comparison of nomograms with the AJCC staging system.

			Nomogram score	8th AJCC stage	p
Training cohort	PDAC	AIC	6734.785	6803.814	
		loglikelihood	-3366.392	-3396.907	
		c-index	0.690(0.667-0.712)	0.652(0.629-0.676)	0.002
	PHC	AIC	3266.077	3307.219	
		loglikelihood	-1632.038	-1648.609	
		c-index	0.669(0.636-0.702)	0.640(0.608-0.672)	0.04
	PBTC	AIC	2649.604	2684.392	
		loglikelihood	-1323.802	-1337.196	
		c-index	0.704(0.672-0.735)	0.656(0.622-0.690)	0.009
Test cohort	PDAC	AIC	2569.793	2625.635	
		loglikelihood	-1283.896	-1307.817	
		c-index	0.669(0.634-0.704)	0.591(0.550-0.631)	<0.0001
	PHC	AIC	1236.191	1266.524	
		loglikelihood	-617.095	-628.262	
		c-index	0.636(0.585-0.688)	0.558(0.502-0.614)	0.0008
	PBTC	AIC	989.924	989.506	
		loglikelihood	-493.962	-489.753	
		c-index	0.643(0.588-0.699)	0.618(0.562-0.674)	0.3

PDAC, pancreatic ductal adenocarcinoma; AIC, Akaike information criterion; PHC, pancreatic head/uncinate ductal adenocarcinoma; PBTC, pancreatic body/tail ductal adenocarcinoma.

### Performance Comparison Between the Nomograms and 8^th^ Edition TNM Staging Systems

**Figure 5:**
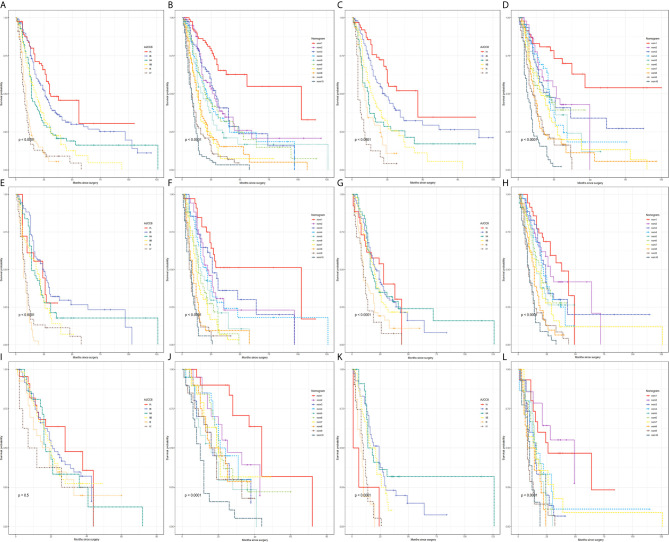
Kaplan–Meier curve analysis of risk classification. Risk classification of the **(A, B)** PDAC group, **(C, D)** PHC group, and **(E, F)** PBTC group in the training cohort. Risk classification of the **(G, H)** PDAC group, **(I, J)** PHC group, and **(K, L)** PBTC group in the test cohort. All log-rank p values for trends were <0.0001, except p=0.5 for **(I)**.

**Figure 6:**
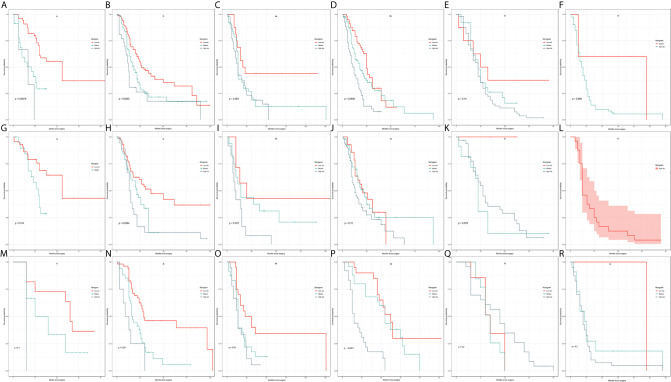
Kaplan–Meier curve analysis of risk stratification. Risk stratification in the training cohort for each 8^th^ AJCC substage in the **(A–F)** PDAC group, **(G–L)** PHC group, and **(M–R)** PBTC group. The log-rank p values were <0.05 for **(A, B, D, G, H, I, N**–**P)**.

**Figure 7:**
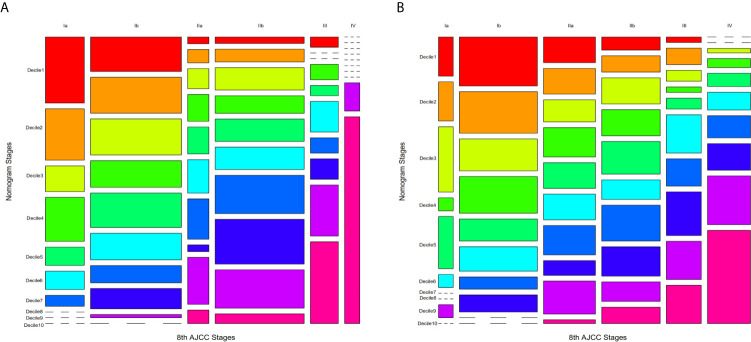
Mosaic plots using the training cohort. **(A)** Mosaic plots for PHC and **(B)** PBTC in which each of the 10 deciles was represented by 1 of 10 consecutive rainbow colors. The area of the individual mosaics represents the represents the proportions of corresponding patients. The short-segmented lines indicate a frequency of zero.

**Figure 8:**
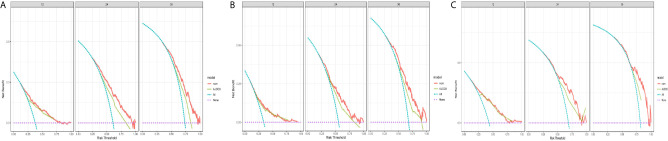
Decision curve analysis and comparison of the nomograms with the 8^th^AJCC stages. Decision curve analysis (DCA) of the nomograms for predicting **(A)** 1-, 2-, and 3-year survival in the PDAC group; **(B)** 1-, 2-, and 3-year survival in the PHC; and **(C)** 1-, 2-, and 3-year survival in the PBTC group.

In comparison to the AJCC 8^TH^ staging system, the nomograms showed greater log-likelihoods and c-indexes, together with smaller values of AIC for CSS in the PDAC, PHC, and PBTC groups (**Table 5**), indicating that the newly established nomograms were more robust for survival prediction than the AJCC stages. Additionally, instead of the six stages classified by 8^th^ AJCC system, the new models stratified patients into 10 nomo stages, providing better discriminative ability (**Figure 5**). As shown in **Suppl. Table 7**, the HRs for the Nomo stages also confirmed the classification ability of the nomograms. Further analysis (**Figure 6**) showed a good ability for risk stratification using the nomograms by stratifying the AJCC 8^th^ stages into the low-, medium-, and high-risk groups. The mosaic plots intuitively demonstrated the dramatic survival heterogeneity between the 8^th^ edition AJCC stages and the nomo stages (**Figure 7**). Finally, the ROC curve showed the superior sensitivity and specificity of nomograms compared with the 8^th^ edition AJCC stages (**Suppl. Figure 1**), and DCA demonstrated that the net benefit was consistently enhanced in all cohorts of the nomograms with wide ranges of threshold probabilities compared with the TNM stages, suggesting the favorable clinical applicability of the nomograms for predicting survival (**Figure 8**).

### Comparative Analysis of the Predictive Performance Among Three Nomograms

The PBTC nomogram had optimal AUCs in both the training cohort and test cohort, while the AUCs for the PDAC nomogram were higher than those of the PHC nomogram (**Table 6**). The aforementioned criteria (c-index) were consistent with the results of ROC curves (**Table 5**), indicating that the nomogram for PBTC performed best and the nomogram for PDAC was more robust for survival prediction compared with that for PHC.

**Table 6:** Time-dependent ROC curve analysis.

	Training cohort AUC (%)						Test cohort AUC (%)					
Study cohort	1 year	95% CI	2 year	95% CI	3 year	95% CI	1 year	95% CI	2 year	95% CI	3 year	95% CI
PDAC	76.30	72.78-79.83	74.72	71.02-78.43	73.16	68.4-77.93	72.07	66.12-78.03	73.81	67.99-79.64	73.39	66.31-80.49
PHC	72.32	67.16-77.5	71.34	66.29-76.39	72.58	66.6-78.57	66.06	57.34-74.79	66.28	57.71-74.85	64.81	54.08-75.54
PBTC	80.00	75.07-84.92	77.33	71.67-82.98	76.97	69.45-84.49	69.69	60.69-78.7	76.63	67.22-86.06	83.70	71.85-95.56

PDAC, pancreatic ductal adenocarcinoma; PHC, pancreatic head/uncinate ductal adenocarcinoma; PBTC, pancreatic body/tail ductal adenocarcinoma.

## Discussion

PDAC can occur in different regions of the pancreas, and the influence of primary cancer location on the prognosis of PDAC has not been fully elucidated. Several nomograms had been established before to demonstrated their superiority over 8^th^AJCC system, some for the PBTC (16, 17), others for the PHC (18). However, this is the first study, to the best of our knowledge, developed three nomograms simultaneously based on the heterogeneous clinicopathological characteristics identified between PHC and PBTC. The novel major findings of this study are summarized as follows: (1) the primary cancer location was an independent factor for prognosis of patients with PDAC after surgery; (2) differential independent risk factors according to different primary tumor locations were identified to be significantly correlated with a poor prognosis; (3) three nomograms for the prediction of prognosis in PDAC, PHC, and PBTC were constructed on the basis of the identified independent prognosis factors; (4) these nomograms performed and calibrated well, with c-indexes of 0.690 (95% CI 0.667–0.712) for PDCA, 0.669 (95% CI 0.636–0.702) for PHC, and 0.704 (95% CI 0.672–0.735) for PBTC; and (5) performance comparison suggested that the newly established nomograms offer greater clinical net benefits than the 8^th^ edition AJCC staging system. As such, these nomograms have the potential to be novel and better approaches for predicting survival of PHC and PBTC patients after surgery.

In the present study, we identified that tumor location was an independent risk factor for poor prognosis in PDAC. The prognosis of patients with PHC was better than that of patients with PBTC. Previous studies have shown the primary tumor location may have a significant impact on prognosis in colorectal and gastric cancer (19, 20), whereas its influence in PDAC remains controversial. Some previous studies have demonstrated that differences existed in the biological and oncological behavior and prognosis between head/uncinate and body/tail PC (6, 7, 21–25), while other studies (9, 26, 27) identified no significant correlation between primary tumor location and OS. Huang et al. (27) analyzed 11,837 patients with chemotherapy and surgical resection from five different countries, indicating that tumor location had no influence on survival. Nevertheless, they recruited the patients of stage I and II from 2003 to 2014, and the AJCC staging system was less accurate in early years. In contrast, we included patients from all stages based on their resectability, and the differences of prognosis between PHC and PBTC mainly occurred in stage Ia and III. It has been recognized that the head of the pancreas and the tail of pancreas arise from different embryonic anlagen, with the anterior domain of the pancreatic head together with the body and tail of pancreas derived from the dorsal primordia,while the ventral primordia formed inferior portions of the pancreatic head and uncinate process. Due to their differential embryological origins and differences in histology and cytology (28, 29), Dreyer et al. (24) reported that tumors in body and tail more likely were of the squamous subtype and were enriched for gene programs associated with tumor invasion and poor antitumor immune response. Hence, worse survival was observed with tumors in PBTC, consistent with the findings of some other studies (3, 18, 30). However, other studies proposed conclusion contrarily (8, 31).

The number of metastatic lymph nodes was not an independent risk factor as compared with other important clinical indicators such as tumor size and LNR. The 8^th^ AJCC staging system overestimated the weight of lymph nodes, and it was inappropriate to classify all N2 stage cases as stage III (32, 33). We further compared the risk factors for PHC and PBTC and obtained some interesting findings. First, PHC and PBTC were found to have some unique independent risk factors, which indicated that their clinical-pathological behaviors might be different. Secondly, tumor size, LNR, tumor differentiation degree, nerve invasion, and distant metastasis were all independent prognostic factors both for PHC and PBTC, which was consistent with previous reports (34–37). Third, the LNR in both groups exhibited independent predictive significance while LNM not. Some studies (38, 39) showed that the LNR had the strongest prediction ability compared with LNC and the 8^th^ N stage. He et al. (40) identified the LNR as an independent predictive factor. Shi et al. (41) and Slidell et al. (42) found that LNC was as important as LNR, especially in node-negative disease. Similarly, in our study, negative lymph nodes were found more often in patients with PBTC, which might explain the strong correlation between LNC and the prognosis of PBTC. Fourth, hyperglycemia was found to an independent risk factor for PHC but not PBTC. Previous research (43–47) had shown that hyperglycemia is associated with worse survival of PC, but only a few studies focused on whether hyperglycemia affects the postoperative prognosis of PDAC. Raghavan et al. (44) reported that the prognosis of PDAC patients with hyperglycemia after surgery is poor, and the mechanism may be related to the Warburg effect. Li et al. (45) suggested that hyperglycemia might correlate with EMT. To date, there has been no report on whether hyperglycemia has distinctive impacts on pancreatic tumors in different locations. The above results demonstrated that the factors for prognosis of PDAC in different regions were heterogeneous, and the ability of 8^th^ AJCC staging system to predict the outcome of PDAC remained mediocre as it defines PHC and PBTC as the same tumor.

We established nomograms on the basis of the differences in independent risk factors for PDAC, PHC and PBTC, and they showed high accuracy and reliability in the prognostic prediction of PHC and PBTC. Notably, our results support that the performance of these nomograms was superior to the latest edition 8^th^ AJCC staging system. Furthermore, the newly established nomograms were able to stratify PDAC into 10 nomo stages in comparison with only three prognostic subgroups by each 8^th^ AJCC stage, achieving more robust risk classification and stratification. Although there were many stages, clinicians only need the scores of patients according to nomograms, and the scores had one-to-one correspondence with the corresponding stage. Given that the better classification and stratification abilities can classify patients more precisely, the nomograms developed in this study may better help clinicians to identify high-risk patients and thereby promote personalized treatment planning. In addition, DCA verified the favorable clinical validity of the nomograms with consistently enhanced net benefits compared with the latest AJCC staging system. Among the three new models, the nomogram for PBTC had the best performance, as evidenced by the highest c-index and AUC value, while the nomogram for PDAC was slightly better than that for PHC. Therefore, we suggest that the nomogram for PDAC can be used in PHC patients to achieve more accurate survival prediction.

The present study has several potential limitations. First, PDAC patients were retrospectively recruited from three medical centers in China, the information of some impactful predictors such as cancer recurrence, neoadjuvant and adjuvant chemotherapy was incomplete, and differences in surgical procedure and postoperative pathological examinations may have existed, these might be the reasons for the moderate c-indexes, and thus, a prospective study is needed to validate the performance of the nomograms. Second, the enrolled patients included mainly individuals of Chinese ethnic population; thus, the nomograms established in this study will need to be verified in other ethnic populations. Third, this study enrolled patients with M1 stage; however, these were the patients with hepatic metastasis who showed a survival benefit following hepatic metastasis resection for PDAC, as reported by two small single-center series (48, 49). Fourth, genetic factors were not integrated into the analysis of risk factors for prognosis, as they might influence the prognosis.

In summary, the present study shows the differential clinical-pathological characteristics and after-surgery outcomes between PHC and PBTC, and demonstrates the prognosis of them should be evaluated by different staging systems, which have been successfully constructed in this study. The results show that these nomograms perform well and are well calibrated, and therefore, they hold potential to be used as novel and improved tools for the prediction of survival among PHC and PBTC patients after surgical treatment.

## Data Availability Statement

The original contributions presented in the study are included in the article/**Supplementary Material**. Further inquiries can be directed to the corresponding authors.

## Ethics Statement

The studies involving human participants were reviewed and approved by the ethics committee of Fujian Medical University Union Hospital, Fujian Provincial Hospital, Ruijin Hospital. The patients/participants provided their written informed consent to participate in this study. Written informed consent was obtained from the individual(s) for the publication of any potentially identifiable images or data included in this article.

## Author Contributions

GL, C-YL and J-ZC: carried out the concepts, design, definition of intellectual content, literature search, data acquisition, data analysis and manuscript preparation. LH, CY, and Y-FT: helped perform the analysis with constructive discussions. QD and Y-TW: provided assistance for data acquisition, data analysis and statistical analysis. SC, QZ, and Y-LC: contributed to the conception of the study and performed manuscript review. All authors contributed to the article and approved the submitted version.

## Funding

This work was supported by the high-level hospital foster grants from Fujian Provincial Hospital (#2019HSJJ13 to SC), the Natural Science Foundation for Distinguished Young Scholars of Fujian Province (#2018J06020 to SC), the Education and Scientific Research Foundation of Fujian Province (#2060402 to SC), the joint Funds for the innovation of science and technology, Fujian Province (#2018Y9098 to SC), the Fujian Provincial Health and Family Planning Research Medical Innovation Project (2019-cx-3 to SC), the Natural Science Foundation of Fujian Province (#2017J01206 to QD), the Startup Fund for scientific research, Fujian Medical University (#2019QH1036 to GL) and Fujian Province Educational Research Project for Youths (#JAT190185 to GL).

## Conflict of Interest

The authors declare that the research was conducted in the absence of any commercial or financial relationships that could be construed as a potential conflict of interest.

## Supplementary Material

Supplementary Figure 1 ROC curve analysis and comparison of the nomograms with the 8thAJCC stages. ROC curve analysis of the nomograms for predicting **(A)** 1-, 2-, and 3-year survival in the PDAC group; **(B)** 1-, 2-, and 3-year survival in the PHC; and **(C)** 1-, 2-, and 3-year survival in the PBTC group.

## References

[1] Bray F Ferlay J Soerjomataram I Siegel RL Torre LA Jemal A. Global Cancer Statistics 2018: GLOBOCAN Estimates of Incidence and Mortality Worldwide for 36 Cancers in 185 Countries. CA Cancer J Clin (2018) 68:394–424. 10.3322/caac.21492 30207593

[2] Neoptolemos JP Palmer DH Ghaneh P Psarelli EE Valle JW Halloran CM. Comparison of Adjuvant Gemcitabine and Capecitabine With Gemcitabine Monotherapy in Patients With Resected Pancreatic Cancer (ESPAC-4): A Multicentre, Open-Label, Randomised, Phase 3 Trial. Lancet (London England) (2017) 389:1011–24. 10.1016/s0140-6736(16)32409-6 28129987

[3] Artinyan A Soriano PA Prendergast C Low T Ellenhorn JD Kim J. The Anatomic Location of Pancreatic Cancer is a Prognostic Factor for Survival. HPB (Oxford) (2008) 10:371–6. 10.1080/13651820802291233 PMC2575681 18982154

[4] Brennan MF Moccia RD Klimstra D. Management of Adenocarcinoma of the Body and Tail of the Pancreas. Ann Surg (1996) 223:506–11; discussion 11-2. 10.1097/00000658-199605000-00006 8651741 PMC1235171

[5] Watanabe I Sasaki S Konishi M Nakagohri T Inoue K Oda T. Onset Symptoms and Tumor Locations as Prognostic Factors of Pancreatic Cancer. Pancreas (2004) 28:160–5. 10.1097/00006676-200403000-00007 15028948

[6] Lau MK Davila JA Shaib YH. Incidence and Survival of Pancreatic Head and Body and Tail Cancers: A Population-Based Study in the United States. Pancreas (2010) 39:458–62. 10.1097/MPA.0b013e3181bd6489 19924019

[7] Zheng Z Wang M Tan C Chen Y Ping J Wang R. Disparities in Survival by Stage After Surgery Between Pancreatic Head and Body/Tail in Patients With Nonmetastatic Pancreatic Cancer. PloS One (2019) 14:e0226726. 10.1371/journal.pone.0226726 31856205 PMC6922472

[8] Winer LK Dhar VK Wima K Morris MC Lee TC Shah SA. The Impact of Tumor Location on Resection and Survival for Pancreatic Ductal Adenocarcinoma. J Surg Res (2019) 239:60–6. 10.1016/j.jss.2019.01.061 30802706

[9] van Erning FN Mackay TM van der Geest LGM Groot Koerkamp B van Laarhoven HWM Bonsing BA. Association of the Location of Pancreatic Ductal Adenocarcinoma (Head, Body, Tail) With Tumor Stage, Treatment, and Survival: A Population-Based Analysis. Acta Oncol (Stockholm Sweden) (2018) 57:1655–62. 10.1080/0284186x.2018.1518593 30264642

[10] Li G Chen JZ Chen S Lin SZ Pan W Meng ZW. Development and Validation of Novel Nomograms for Predicting the Survival of Patients After Surgical Resection of Pancreatic Ductal Adenocarcinoma. Cancer Med (2020) 9:3353–70. 10.1002/cam4.2959 PMC7221449 32181599

[11] Harrell F. Regression Modeling Strategies: With Applications to Linear Models, Logistic and Ordinal Regression, and Survival Analysis. Verlag New York: Springer (2002).

[12] Kang L Chen W Petrick NA Gallas BD. Comparing Two Correlated C Indices With Right-Censored Survival Outcome: A One-Shot Nonparametric Approach. Stat Med (2015) 34:685–703. 10.1002/sim.6370 25399736 PMC4314453

[13] Hung H CC. Estimation Methods for Time-Dependent AUC Models With Survival Data. Can J Stat (2010) 38:8–26. 10.2307/27805213

[14] Vickers AJ Elkin EB. Decision Curve Analysis: A Novel Method for Evaluating Prediction Models. Med Decis Making (2006) 26:565–74. 10.1177/0272989x06295361 PMC2577036 17099194

[15] Allen PJ Kuk D Castillo CF Basturk O Wolfgang CL Cameron JL. Multi-Institutional Validation Study of the American Joint Commission on Cancer (8th Edition) Changes for T and N Staging in Patients With Pancreatic Adenocarcinoma. Ann Surg (2017) 265:185–91. 10.1097/sla.0000000000001763 PMC5611666 27163957

[16] He C Sun S Zhang Y Lin X Li S. Score for the Overall Survival Probability of Patients With Pancreatic Adenocarcinoma of the Body and Tail After Surgery: A Novel Nomogram-Based Risk Assessment. Front Oncol (2020) 10:590. 10.3389/fonc.2020.00590 32426278 PMC7212341

[17] Zou Y Shi N Ruan S Jin L Yin Z Hang H. Development of a Nomogram to Predict Diseasespecific Survival for Patients After Resection of a non-Metastatic Adenocarcinoma of the Pancreatic Body and Tail. Front Oncol (2020) 10:526602. 10.3389/fonc.2020.526602 33194585 PMC7658586

[18] Li H-b Zhou J Zhao F-q. A Prognostic Nomogram for Disease-Specific Survival in Patients With Pancreatic Ductal Adenocarcinoma of the Head of the Pancreas Following Pancreaticoduodenectomy. Med Sci Monit (2018) 24:6313–21. 10.12659/MSM.909649 PMC6144730 30198517

[19] Petrelli F Ghidini M Barni S Steccanella F Sgroi G Passalacqua R. Prognostic Role of Primary Tumor Location in non-Metastatic Gastric Cancer: A Systematic Review and Meta-Analysis of 50 Studies. Ann Surg Oncol (2017) 24:2655–68. 10.1245/s10434-017-5832-4 28299508

[20] Petrelli F Tomasello G Borgonovo K Ghidini M Turati L Dallera P. Prognostic Survival Associated With Left-Sided vs Right-Sided Colon Cancer: A Systematic Review and Meta-Analysis. JAMA Oncol (2017) 3:211–9. 10.1001/jamaoncol.2016.4227 27787550

[21] Birnbaum DJ Bertucci F Finetti P Birnbaum D Mamessier E. Head and Body/Tail Pancreatic Carcinomas are Not the Same Tumors. Cancers (2019) 11:497. 10.3390/cancers11040497 PMC6520848 30965637

[22] Mackay TM van Erning FN van der Geest LGM de Groot JWB Haj Mohammad N Lemmens VE. Association Between Primary Origin (Head, Body and Tail) of Metastasised Pancreatic Ductal Adenocarcinoma and Oncologic Outcome: A Population-Based Analysis. Eur J Cancer (Oxford Engl 1990) (2019) 106:99–105. 10.1016/j.ejca.2018.10.008 30476732

[23] Yin L Xiao L Gao Y Wang G Gao H Peng Y. Comparative Bioinformatical Analysis of Pancreatic Head Cancer and Pancreatic Body/Tail Cancer. Med Oncol (Northwood London England) (2020) 37:46. 10.1007/s12032-020-01370-0 32277286

[24] Dreyer SB Jamieson NB Upstill-Goddard R Bailey PJ McKay CJ Biankin AV. Defining the Molecular Pathology of Pancreatic Body and Tail Adenocarcinoma. Br J Surg (2018) 105:e183–e91. 10.1002/bjs.10772 PMC5817249 29341146

[25] Lee M Kwon W Kim H Byun Y Han Y Kang JS. The Role of Location of Tumor in the Prognosis of the Pancreatic Cancer. Cancers (2020) 12:2036. 10.3390/cancers12082036 PMC7465041 32722142

[26] Ruess DA Makowiec F Chikhladze S Sick O Riediger H Hopt UT. The Prognostic Influence of Intrapancreatic Tumor Location on Survival After Resection of Pancreatic Ductal Adenocarcinoma. BMC Surg (2015) 15:123. 10.1186/s12893-015-0110-5 26615588 PMC4663036

[27] Huang L Balavarca Y van der Geest L Lemmens V Van Eycken L De Schutter H. Development and Validation of a Prognostic Model to Predict the Prognosis of Patients Who Underwent Chemotherapy and Resection of Pancreaticadenocarcinoma: A Large International Population-Based Cohort Study. BMC Med (2019) 17:66. 10.1186/s12916-019-1304-y 30905320 PMC6432746

[28] Radi M Gaubert J Cristol-Gaubert R Baecker V Travo P Prudhomme M. A 3D Reconstruction of Pancreas Development in the Human Embryos During Embryonic Period (Carnegie Stages 15-23). Surg Radiol Anat (2010) 32:11–5. 10.1007/s00276-009-0533-8 19921091

[29] Tadokoro H Kozu T Toki F Kobayashi M Hayashi N. Embryological Fusion Between the Ducts of the Ventral and Dorsal Primordia of the Pancreas Occurs in Two Manners. Pancreas (1997) 14:407–14. 10.1097/00006676-199705000-00012 9163788

[30] Sheng W Dong M Wang G Shi X Gao W Wang K. The Diversity Between Curatively Resected Pancreatic Head and Body-Tail Cancers Based on the 8th Edition of AJCC Staging System: A Multicenter Cohort Study. BMC Cancer (2019) 19:981. 10.1186/s12885-019-6178-z 31640615 PMC6805668

[31] Meng Z Cao M Zhang Y Liu Z Wu S Wu H. Tumor Location as an Indicator of Survival in T1 Resectable Pancreatic Ductal Adenocarcinoma: A Propensity Score-Matched Analysis. BMC Gastroenterol (2019) 19:59. 10.1186/s12876-019-0975-3 31014264 PMC6480875

[32] Shin DW Lee JC Kim J Woo SM Lee WJ Han SS. Validation of the American Joint Committee on Cancer 8th Edition Staging System for the Pancreatic Ductal Adenocarcinoma. Eur J Surg Oncol (2019) 45:2159–65. 10.1016/j.ejso.2019.06.002 31202572

[33] Song M Yoon SB Lee IS Hong TH Choi HJ Choi MH. Evaluation of the Prognostic Value of the New AJCC 8th Edition Staging System for Patients With Pancreatic Adenocarcinoma; A Need to Subclassify Stage III? Eur J Cancer (Oxford Engl 1990) (2018) 104:62–9. 10.1016/j.ejca.2018.08.027 30326370

[34] Yu FJ Shih HY Wu CY Chuang YS Lee JY Li HP. Enteral Nutrition and Quality of Life in Patients Undergoing Chemoradiotherapy for Esophageal Carcinoma: A Comparison of Nasogastric Tube, Esophageal Stent, and Ostomy Tube Feeding. Gastrointest Endosc (2018) 88:21–31.e4. 10.1016/j.gie.2017.11.030 29225081

[35] Pindak D Tomas M Dolnik J Duchon R Pavlendova J. Morbidity, Mortality and Long Term Survival in Patients With Vascular Resection in Pancreatic Cancer - Single Center Experience. Neoplasma (2017) 64:460–3. 10.4149/neo_2017_318 28253726

[36] Yamamoto T Yagi S Kinoshita H Sakamoto Y Okada K Uryuhara K. Long-Term Survival After Resection of Pancreatic Cancer: A Single-Center Retrospective Analysis. World J Gastroenterol (2015) 21:262–8. 10.3748/wjg.v21.i1.262 PMC4284344 25574100

[37] Schorn S Demir IE Haller B Scheufele F Reyes CM Tieftrunk E. The Influence of Neural Invasion on Survival and Tumor Recurrence in Pancreatic Ductal Adenocarcinoma - A Systematic Review and Meta-Analysis. Surg Oncol (2017) 26:105–15. 10.1016/j.suronc.2017.01.007 28317579

[38] Riediger H Keck T Wellner U zur Hausen A Adam U Hopt UT. The Lymph Node Ratio is the Strongest Prognostic Factor After Resection of Pancreatic Cancer. J Gastrointest Surg (2009) 13:1337–44. 10.1007/s11605-009-0919-2 19418101

[39] Pawlik TM Gleisner AL Cameron JL Winter JM Assumpcao L Lillemoe KD. Prognostic Relevance of Lymph Node Ratio Following Pancreaticoduodenectomy for Pancreatic Cancer. Surgery (2007) 141:610–8. 10.1016/j.surg.2006.12.013 17462460

[40] He C Zhang Y Cai Z Lin X Li S. Overall Survival and Cancer-Specific Survival in Patients With Surgically Resected Pancreatic Head Adenocarcinoma: A Competing Risk Nomogram Analysis. J Cancer (2018) 9:3156–67. 10.7150/jca.25494 PMC6134825 30210639

[41] Shi S Hua J Liang C Meng Q Liang D Xu J. Proposed Modification of the 8th Edition of the AJCC Staging System for Pancreatic Ductal Adenocarcinoma. Ann Surg (2019) 269:944–50. 10.1097/sla.0000000000002668 29334560

[42] Slidell MB Chang DC Cameron JL Wolfgang C Herman JM Schulick RD. Impact of Total Lymph Node Count and Lymph Node Ratio on Staging and Survival After Pancreatectomy for Pancreatic Adenocarcinoma: A Large, Population-Based Analysis. Ann Surg Oncol (2008) 15:165–74. 10.1245/s10434-007-9587-1 17896141

[43] Liao WC Tu YK Wu MS Lin JT Wang HP Chien KL. Blood Glucose Concentration and Risk of Pancreatic Cancer: Systematic Review and Dose-Response Meta-Analysis. BMJ (Clinical Res ed) (2015) 350:g7371. 10.1136/bmj.g7371 PMC4282179 25556126

[44] Raghavan SR Ballehaninna UK Chamberlain RS. The Impact of Perioperative Blood Glucose Levels on Pancreatic Cancer Prognosis and Surgical Outcomes: An Evidence-Based Review. Pancreas (2013) 42:1210–7. 10.1097/MPA.0b013e3182a6db8e 24152946

[45] Li W Zhang L Chen X Jiang Z Zong L Ma Q. Hyperglycemia Promotes the Epithelial-Mesenchymal Transition of Pancreatic Cancer Via Hydrogen Peroxide. Oxid Med Cell Longev (2016) 2016:5190314. 10.1155/2016/5190314 27433288 PMC4940572

[46] Sharma A Smyrk TC Levy MJ Topazian MA Chari ST. Fasting Blood Glucose Levels Provide Estimate of Duration and Progression of Pancreatic Cancer Before Diagnosis. Gastroenterology (2018) 155:490–500.e2. 10.1053/j.gastro.2018.04.025 29723506 PMC6067966

[47] Nagai M Murakami Y Tamakoshi A Kiyohara Y Yamada M Ukawa S. Fasting But Not Casual Blood Glucose is Associated With Pancreatic Cancer Mortality in Japanese: EPOCH-JAPAN. Cancer Causes Control (2017) 28:625–33. 10.1007/s10552-017-0884-0 28352981

[48] Shrikhande SV Kleeff J Reiser C Weitz J Hinz U Esposito I. Pancreatic Resection for M1 Pancreatic Ductal Adenocarcinoma. Ann Surg Oncol (2007) 14:118–27. 10.1245/s10434-006-9131-8 17066229

[49] Yamada H Hirano S Tanaka E Shichinohe T Kondo S. Surgical Treatment of Liver Metastases From Pancreatic Cancer. HPB (Oxford) (2006) 8:85–8. 10.1080/13651820500472200 PMC2131410 18333251

