# Supplementary figures and images for: Construction and Validation of Novel Nomograms for Predicting Prognosis of Pancreatic Ductal Adenocarcinoma After Surgery According to Different Primary Cancer Locations
Source: Front Oncol. 2021 Apr 23;11:646082. doi: 10.3389/fonc.2021.646082 (PMC8103839; doi:10.3389/fonc.2021.646082)

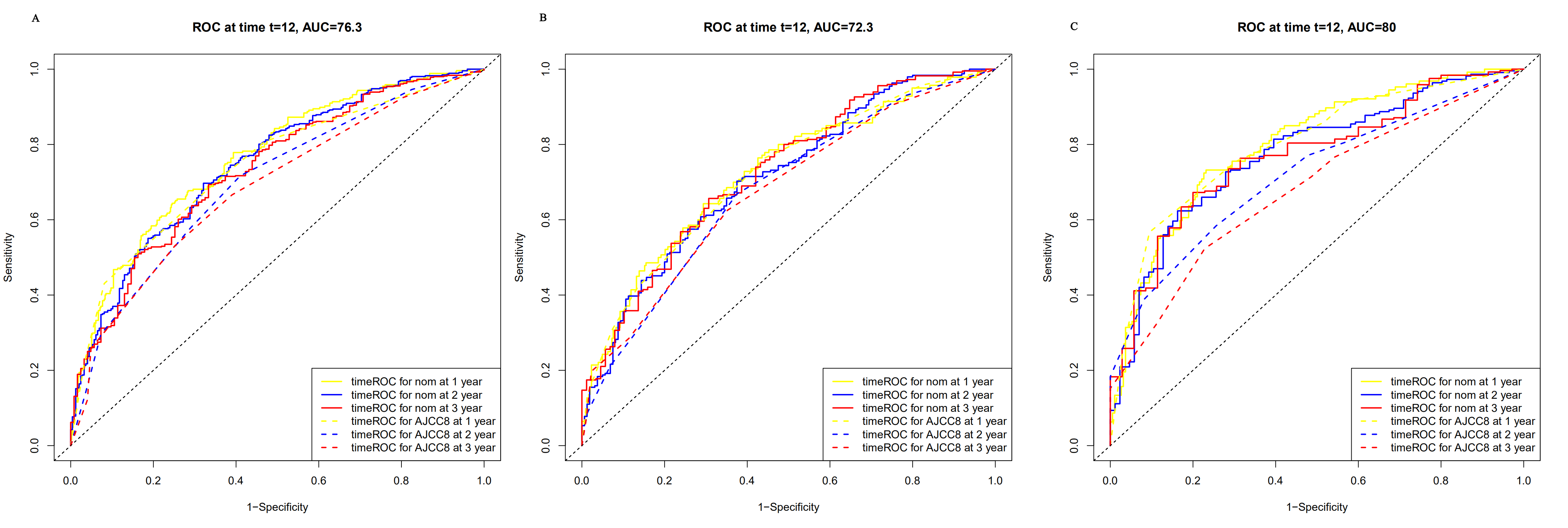

Supplement: Supplementary Figure 1 — ROC curve analysis and comparison of the nomograms with the 8thAJCC stages. ROC curve analysis of the nomograms for predicting (A) 1-, 2-, and 3-year survival in the PDAC group; (B) 1-, 2-, and 3-year survival in the PHC; and (C) 1-, 2-, and 3-year survival in the PBTC group. [file Image_1.tif]
