# Supplementary material for: Construction and Validation of Novel Nomograms for Predicting Prognosis of Pancreatic Ductal Adenocarcinoma After Surgery According to Different Primary Cancer Locations
Source: Front Oncol. 2021 Apr 23;11:646082. doi: 10.3389/fonc.2021.646082 (PMC8103839; doi:10.3389/fonc.2021.646082)
Supplement: Supplementary file 2 [file Table_1.doc]

**Suppl. Table 1 Baseline demographic and clinical characteristics of the PHC patients**

| Variables | Training cohort |  | Test cohort |  | P value |
| --- | --- | --- | --- | --- | --- |
|  | n=467 |  | n=199 |  |  |
| Gender, n, (%) |  |  |  |  | 0.482 |
| Female | 165 | (35.33) | 76 | (38.19) |  |
| Male | 302 | (64.67) | 123 | (61.81) |  |
| Age (years), median, IQR | 62.00 | 56.00, 69.00 | 61.00 | 57.00, 69.00 | 0.922 |
| BMI (kg/m^2^), mean, sd | 22.88 | 3.44 | 22.61 | 3.55 | 0.356 |
| Symptoms, (%) |  |  |  |  | 0.984 |
| Yes (including abdominal pain/gastrointestinal symptoms) | 350 | (74.95) | 149 | (74.87) |  |
| No | 117 | (25.05) | 50 | (25.13) |  |
| TBIL (umol/L), mean, sd | 97.24 | 94.03 | 110.20 | 103.29 | 0.114 |
| ALB (g/L), mean, sd | 36.58 | 5.25 | 36.64 | 4.75 | 0.892 |
| Fasting blood glucose (mmol/L), mean, sd | 6.42 | 2.24 | 6.85 | 2.53 | 0.032 |
| CA125, (%) |  |  |  |  | 0.585 |
| Normal | 396 | (84.80) | 172 | (86.43) |  |
| Elevated | 71 | (15.20) | 27 | (13,57) |  |
| CA19-9, (%) |  |  |  |  | 0.148 |
| Normal | 108 | (23.13) | 36 | (18.09) |  |
| Elevated | 359 | (76.87) | 163 | (81.91) |  |
| CEA, (%) |  |  |  |  | 0.027 |
| Normal | 351 | (75.16) | 133 | (66.83) |  |
| Elevated | 116 | (24.84) | 66 | (33.17) |  |
| Smoking history, (%) |  |  |  |  | 0.686 |
| Yes | 122 | (26.12) | 55 | (27.64) |  |
| No | 345 | (73.88) | 144 | (72.36) |  |
| Drinking history, (%) |  |  |  |  | 0.238 |
| Yes | 87 | (18.63) | 45 | (22.61) |  |
| No | 380 | (81.37) | 154 | (77.39) |  |
| Histology, n, (%) |  |  |  |  | 0.784 |
| Well differentiated | 18 | (3.85) | 8 | (4.02) |  |
| Moderately differentiated | 232 | (49.68) | 93 | (46.73) |  |
| Poorly differentiated | 217 | (46.47) | 98 | (49.25) |  |
| Intraoperative blood loss (ml), mean, sd | 613.19 | 488.26 | 575.98 | 437.92 | 0.354 |
| Tumor size (cm), mean, sd | 3.23 | 1.30 | 3.36 | 1.43 | 0.267 |
| Perineuronal invasion n, (%) |  |  |  |  | 0.366 |
| Yes | 295 | (63.17) | 133 | (66.83) |  |
| No | 172 | (36.83) | 66 | (33.17) |  |
| pT stage, n, (%) |  |  |  |  | 0.524 |
| pT1 | 102 | (21.84) | 44 | (22.11) |  |
| pT2 | 281 | (60.17) | 110 | (55.28) |  |
| pT3 | 77 | (16.49) | 42 | (21.11) |  |
| pT4 | 7 | (1.50) | 3 | (1.51) |  |
| pN stage, n, (%) |  |  |  |  | 0.529 |
| N0 | 261 | (55.89) | 108 | (54.27) |  |
| N1 | 161 | (34.48) | 66 | (33.17) |  |
| N2 | 45 | (9.64) | 25 | (12.56) |  |
| LNC, mean, sd | 11.27 | 8.51 | 12.29 | 8.61 | 0.157 |
| LNR, mean, sd | 0.11 | 0.19 | 0.12 | 0.20 | 0.699 |
| LNM, mean sd | 1.16 | 1.96 | 1.25 | 2.13 | 0.578 |
| Metastasis, n, (%) |  |  |  |  | 0.468 |
| M0 | 442 | (94.65) | 191 | (95.98) |  |
| M1 | 25 | (5.35) | 8 | (4.02) |  |
| 8^th^ AJCC stage, n, (%) |  |  |  |  | 0.444 |
| Ia | 64 | (13.70) | 22 | (11.06) |  |
| Ib | 150 | (32.12) | 60 | (30.15) |  |
| IIa | 35 | (7.49) | 21 | (10.55) |  |
| IIb | 147 | (31.48) | 61 | (30.65) |  |
| III | 46 | (9.85) | 27 | (13.57) |  |
| IV | 25 | (5.35) | 8 | (4.02) |  |
| Neoadjuvant chemotherapy, n, (%) |  |  |  |  | 0.774 |
| Yes | 131 | (28.05) | 58 | (29.15) |  |
| No | 336 | (71.95) | 141 | (70.85) |  |

Note: PHC, pancreatic head/uncinate ductal adenocarcinoma; IQR, interquartile range; SD, standard deviation; BMI, body mass index; TBIL, total bilirubin; ALB, albumin; LNC, lymph node count; LNM, lymph node metastasis; LNR, lymph node ratio; CA125, cancer antigen 125; CA19-9, cancer antigen 19-9; CEA, carcino-embryonic antigen.

**Suppl. Table 2** Baseline demographic and clinical characteristics of the PBTC patients

| Variables | Training cohort |  | Test cohort |  | P value |
| --- | --- | --- | --- | --- | --- |
|  | n=346 |  | n=148 |  |  |
| Gender, n, (%) |  |  |  |  | 0.129 |
| Female | 166 | (47.98) | 60 | (40.54) |  |
| Male | 180 | (52.02) | 88 | (59.46) |  |
| Age (years), median, IQR | 63.00 | 57.00, 70.00 | 62.00 | 57.00, 68.00 | 0.143 |
| BMI (kg/m^2^), mean, sd | 22.83 | 3.20 | 22.96 | 3.09 | 0.666 |
| Symptoms, (%) |  |  |  |  | 0.208 |
| Yes (including abdominal pain/gastrointestinal symptoms) | 218 | (63.01) | 102 | (68.92) |  |
| No | 128 | (36.99) | 46 | (31.08) |  |
| TBIL (umol/L), mean, sd | 15.28 | 5.90 | 16.17 | 5.88 | 0.126 |
| ALB (g/L), mean, sd | 39.42 | 4.32 | 39.86 | 3.72 | 0.276 |
| Fasting blood glucose (mmol/L), mean, sd | 6.85 | 4.99 | 6.76 | 2.84 | 0.843 |
| CA125, (%) |  |  |  |  | 0.639 |
| Normal | 264 | (76.30) | 110 | (74.32) |  |
| Elevated | 82 | (23.70) | 38 | (25.68) |  |
| CA19-9, (%) |  |  |  |  | 0.643 |
| Normal | 73 | (21.10) | 34 | (22.97) |  |
| Elevated | 273 | (78.90) | 114 | (77.03) |  |
| CEA, (%) |  |  |  |  | 0.367 |
| Normal | 216 | (62.43) | 86 | (58.11) |  |
| Elevated | 130 | (37.57) | 62 | (41.89) |  |
| Smoking history, (%) |  |  |  |  | 0.205 |
| Yes | 84 | (24.28) | 44 | (29.73) |  |
| No | 262 | (75.72) | 104 | (70.27) |  |
| Drinking history, (%) |  |  |  |  | 0.027 |
| Yes | 69 | (19.94) | 43 | (29.05) |  |
| No | 277 | (80.06) | 105 | (70.95) |  |
| Histology, n, (%) |  |  |  |  | 0.569 |
| Well differentiated | 44 | (12.72) | 22 | (14.86) |  |
| Moderately differentiated | 149 | (43.06) | 55 | (37.16) |  |
| Poorly differentiated | 153 | (44.22) | 71 | (47.97) |  |
| Intraoperative blood loss (ml), mean, sd | 505.00 | 487.05 | 529.59 | 428.57 | 0.595 |
| Tumor size (cm), mean, sd | 4.44 | 1.88 | 4.55 | 2.04 | 0.557 |
| Perineuronal invasion n, (%) |  |  |  |  | 0.005 |
| Yes | 207 | (59.83) | 108 | (72.97) |  |
| No | 139 | (40.17) | 40 | (27.03) |  |
| pT stage, n, (%) |  |  |  |  | 0.523 |
| pT1 | 25 | (7.23) | 6 | (4.05) |  |
| pT2 | 163 | (47.11) | 71 | (47.97) |  |
| pT3 | 120 | (34.68) | 51 | (34.46) |  |
| pT4 | 38 | (10.98) | 20 | (13.51) |  |
| pN stage, n, (%) |  |  |  |  | 0.938 |
| N0 | 219 | (63.29) | 95 | (64.19) |  |
| N1 | 108 | (31.21) | 46 | (31.08) |  |
| N2 | 19 | (5.49) | 7 | (4.73) |  |
| LNC, mean, sd | 4.86 | 5.17 | 4.94 | 5.06 | 0.882 |
| LNR, mean, sd | 0.18 | 0.32 | 0.17 | 0.33 | 0.849 |
| LNM, mean sd | 0.92 | 2.29 | 0.79 | 1.74 | 0.551 |
| Metastasis, n, (%) |  |  |  |  | 0.747 |
| M0 | 293 | (84.68) | 127 | (85.81) |  |
| M1 | 53 | (15.32) | 21 | (14.19) |  |
| 8^th^ AJCC stage, n, (%) |  |  |  |  | 0.773 |
| Ia | 18 | (5.20) | 4 | (2.70) |  |
| Ib | 96 | (27.75) | 41 | (27.70) |  |
| IIa | 64 | (18.50) | 25 | (16.89) |  |
| IIb | 72 | (20.81) | 36 | (24.32) |  |
| III | 43 | (12.43) | 21 | (14.19) |  |
| IV | 53 | (15.32) | 21 | (14.19) |  |
| Neoadjuvant chemotherapy, n, (%) |  |  |  |  | 0.521 |
| Yes | 101 | (29.19) | 39 | (26.35) |  |
| No | 245 | (70.81) | 109 | (73.65) |  |

Note: PBTC, pancreatic body/tail ductal adenocarcinoma; IQR, interquartile range; SD, standard deviation; BMI, body mass index; TBIL, total bilirubin; ALB, albumin; LNC, lymph node count; LNM, lymph node metastasis; LNR, lymph node ratio; CA125, cancer antigen 125; CA19-9, cancer antigen 19-9; CEA, carcino-embryonic antigen.

**Suppl.Table 3** Baseline demographic and clinical characteristics of PDAC patients with 8^th^ AJCC stage Ia disease

| Variables | Head/Uncinate | | Body/Tail |  | p |
| --- | --- | --- | --- | --- | --- |
|  | n=64 |  | n=18 |  |  |
| Gender, n, (%) |  |  |  |  | 0.362 |
| Female | 21 | (32.81) | 8 | (44.44) |  |
| Male | 43 | (67.19) | 10 | (55.56) |  |
| Age (years), median, IQR | 62.50 | 57.75, 69.00 | 66.50 | 59.00, 68.75 | 0.215 |
| BMI (kg/m^2^), mean, SD | 22.47 | 2.66 | 23.00 | 1.96 | 0.442 |
| Symptoms, (%) |  |  |  |  | 0.030 |
| Yes (including abdominal pain/gastrointestinal symptoms) | 46 | (71.88) | 8 | (44.44) |  |
| No | 18 | (28.12) | 10 | (55.56) |  |
| TBIL(umol/L), mean, SD | 97.67 | 84.36 | 14.16 | 4.04 | <0.001 |
| ALB (g/L), mean, SD | 36.70 | 6.15 | 38.53 | 3.32 | 0.230 |
| Fasting blood glucose (mmol/L), mean, SD | 5.93 | 1.81 | 6.70 | 2.91 | 0.175 |
| CA125, (%) |  |  |  |  | 0.584 |
| Normal | 51 | (79.69) | 16 | (88.89) |  |
| Elevated | 13 | (20.31) | 2 | (11.11) |  |
| CA19-9, (%) |  |  |  |  | 0.652 |
| Normal | 20 | (31.25) | 4 | (22.22) |  |
| Elevated | 44 | (68.75) | 14 | (77.78) |  |
| CEA, (%) |  |  |  |  | 1.000 |
| Normal | 52 | (81.25) | 15 | (83.33) |  |
| Elevated | 12 | (18.75) | 3 | (16.67) |  |
| Smoking history, (%) |  |  |  |  | 1.000 |
| Yes | 20 | (31.25) | 6 | (33.33) |  |
| No | 44 | (68.75) | 12 | (66.67) |  |
| Drinking history, (%) |  |  |  |  | 1.000 |
| Yes | 17 | (26.56) | 5 | (27.78) |  |
| No | 47 | (73.44) | 13 | (72.22) |  |
| Histology, n, (%) |  |  |  |  | 0.009 |
| Well differentiated | 4 | (6.25) | 1 | (5.56) |  |
| Moderately differentiated | 35 | (54.69) | 3 | (16.67) |  |
| Poorly differentiated | 25 | (39.06) | 14 | (77.78) |  |
| Intraoperative blood loss (ml), mean, SD | 539.06 | 277.96 | 558.89 | 457.00 | 0.819 |
| Tumor size (cm), mean, SD | 1.82 | 0.30 | 1.76 | 0.30 | 0.406 |
| Perineuronal invasion n, (%) |  |  |  |  | 0.404 |
| Yes | 32 | (50.00) | 11 | (61.11) |  |
| No | 32 | (50.00) | 7 | (38.89) |  |
| LNC, mean, SD | 10.08 | 7.46 | 3.72 | 3.41 | 0.001 |
| Neoadjuvant chemotherapy, n, (%) |  |  |  |  | 0.121 |
| Yes | 23 | (35.94) | 3 | (16.67) |  |
| No | 41 | (64.06) | 15 | (83.33) |  |

Note: PDAC, pancreatic ductal adenocarcinoma; IQR, interquartile range; SD, standard deviation; BMI, body mass index; TBIL, total bilirubin; ALB, albumin; LNC, lymph node count; LNM, lymph node metastasis; LNR, lymph node ratio; CA125, cancer antigen 125; CA19-9, cancer antigen 19-9; CEA, carcino-embryonic antigen.

**Suppl.Table 4 Baseline demographic and clinical characteristics of PDAC patients with 8^th^ AJCC stage III disease**

| Variables | Head/Uncinate |  | Body/Tail |  | p |
| --- | --- | --- | --- | --- | --- |
|  | n=46 |  | n=43 |  |  |
| Gender, n, (%) |  |  |  |  | 0.016 |
| Female | 14 | (30.43) | 24 | (55.81) |  |
| Male | 32 | (69.57) | 19 | (44.19) |  |
| Age (years), median, IQR | 64.00 | 56.00, 67.00 | 61.00 | 54.50, 66.50 | 0.774 |
| BMI (kg/m^2^), mean, SD | 22.20 | 3.06 | 21.38 | 2.99 | 0.206 |
| Symptoms, (%) |  |  |  |  | 0.398 |
| Yes (including abdominal pain/gastrointestinal symptoms) | 34 | (73.91) | 35 | (81.40) |  |
| No | 12 | (26.09) | 8 | (18.60) |  |
| TBIL (umol/L), mean, SD | 99.50 | 112.24 | 15.95 | 6.87 | <0.001 |
| ALB (g/L), mean, SD | 36.52 | 4.53 | 39.41 | 4.73 | 0.004 |
| Fasting blood glucose (mmol/L), mean, SD | 5.94 | 2.22 | 6.02 | 1.37 | 0.839 |
| CA125, (%) |  |  |  |  | 0.064 |
| Normal | 37 | (80.43) | 27 | (62.79) |  |
| Elevated | 9 | (19.57) | 16 | (37.21) |  |
| CA19-9, (%) |  |  |  |  | 0.074 |
| Normal | 15 | (32.61) | 7 | (16.28) |  |
| Elevated | 31 | (67.39) | 36 | (83.72) |  |
| CEA, (%) |  |  |  |  | 0.002 |
| Normal | 41 | (89.13) | 26 | (60.47) |  |
| Elevated | 5 | (10.87) | 17 | (39.53) |  |
| Smoking history, (%) |  |  |  |  | 0.793 |
| Yes | 14 | (30.43) | 12 | (27.91) |  |
| No | 32 | (69.57) | 31 | (72.09) |  |
| Drinking history, (%) |  |  |  |  | 0.889 |
| Yes | 8 | (17.39) | 7 | (16.28) |  |
| No | 38 | (82.61) | 36 | (83.72) |  |
| Histology, n, (%) |  |  |  |  | 0.077 |
| Well differentiated | 0 | (0.00) | 4 | (9.30) |  |
| Moderately differentiated | 20 | (43.48) | 14 | (32.56) |  |
| Poorly differentiated | 26 | (56.52) | 25 | (58.14) |  |
| Intraoperative blood loss (ml), mean, SD | 613.70 | 588.34 | 529.07 | 428.06 | 0.443 |
| Tumor size (cm), mean, SD | 3.55 | 1.56 | 4.62 | 1.91 | 0.004 |
| Perineuronal invasion n, (%) |  |  |  |  | 0.224 |
| Yes | 39 | (84.78) | 32 | (74.42) |  |
| No | 7 | (15.22) | 11 | (25.58) |  |
| pT stage, n, (%) |  |  |  |  | <0.001 |
| pT1 | 6 | (13.04) | 2 | (4.65) |  |
| pT2 | 24 | (52.17) | 6 | (13.95) |  |
| pT3 | 10 | (21.74) | 7 | (16.28) |  |
| pT4 | 6 | (13.04) | 28 | (65.12) |  |
| pN stage, n, (%) |  |  |  |  | <0.001 |
| N0 | 4 | (8.70) | 16 | (37.21) |  |
| N1 | 1 | (2.17) | 12 | (27.91) |  |
| N2 | 41 | (89.13) | 15 | (34.88) |  |
| LNC, mean, SD | 18.35 | (11.82) | 8.67 | (7.58) | <0.001 |
| LNR, mean, SD | 0.36 | (0.26) | 0.29 | (0.39) | 0.306 |
| LNM, mean, SD | 5.41 | (3.04) | 3.19 | (5.20) | 0.015 |
| Neoadjuvant chemotherapy, n, (%) |  |  |  |  | 0.829 |
| Yes | 14 | (30.43) | 14 | (32.56) |  |
| No | 32 | (69.57) | 29 | (67.44) |  |

Note: PDAC, pancreatic ductal adenocarcinoma; IQR, interquartile range; SD, standard deviation; BMI, body mass index; TBIL, total bilirubin; ALB, albumin; LNC, lymph node count; LNM, lymph node metastasis; LNR, lymph node ratio; CA125, cancer antigen 125; CA19-9, cancer antigen 19-9; CEA, carcino-embryonic antigen.

**Suppl. Table 5 Points for categorical variables in nomograms**

| PDAC | | PHC | | PBTC | |
| --- | --- | --- | --- | --- | --- |
| Variables |  | Variables |  | Variables |  |
| Tumor location | Points | Gender | Points | Symptoms | Points |
| Head | 0 | Female | 0 | No | 0 |
| Body/Tail | 9 | Male | 8 | Yes | 13 |
| Gender | Points | CA125 | Points | CEA | Points |
| Female | 0 | Normal | 0 | Normal | 0 |
| Male | 10 | Elevated | 7 | Elevated | 11 |
| Symptoms | Points | CA19-9 | Points | Smoking history |  |
| No | 0 | Normal | 0 | No | 0 |
| Yes | 8 | Elevated | 14 | Yes | 14 |
| CA125 | Points | Histology | Points | Histology | Points |
| Normal | 0 | Well differentiated | 0 | Well differentiated | 0 |
| Elevated | 7 | Moderately differentiated | 5 | Moderately differentiated | 9 |
| CA19-9 | Points | Poorly differentiated | 7 | Poorly differentiated | 30 |
| Normal | 0 | Perineuronal invasion | Points | Perineuronal invasion | Points |
| Elevated | 11 | No | 0 | No | 0 |
| CEA | Points | Yes | 11 | Yes | 18 |
| Normal | 0 | Metastasis | Points | Metastasis | Points |
| Elevated | 8 | M0 | 0 | M0 | 0 |
| Histology | Points | M1 | 32 | M1 | 36 |
| Well differentiated | 0 |  |  |  |  |
| Moderately differentiated | 3 |  |  |  |  |
| Poorly differentiated | 16 |  |  |  |  |
| Perineuronal invasion | Points |  |  |  |  |
| No | 0 |  |  |  |  |
| Yes | 13 |  |  |  |  |
| Metastasis | Points |  |  |  |  |
| M0 | 0 |  |  |  |  |
| M1 | 29 |  |  |  |  |
| Neoadjuvant chemotherapy | Points |  |  |  |  |
| Yes | 0 |  |  |  |  |
| No | 10 |  |  |  |  |

Note: PDAC, pancreatic ductal adenocarcinoma; PHC, pancreatic head/uncinate ductal adenocarcinoma; PBTC, pancreatic body/tail ductal adenocarcinoma; BMI, body mass index; TBIL, total bilirubin; ALB, albumin; LNC, lymph node count; LNM, lymph node metastasis; LNR, lymph node ratio; CA125, cancer antigen 125; CA19-9, cancer antigen 19-9; CEA, carcino-embryonic antigen.

**Suppl. Table 6 Points for continuous variables in the nomograms**

| Variables | Points | | | Variables | Points | Variables | Points | | | Variables | Points | | | Variables | Points | | Variables | Points | | Variables | Points | Variables | Points |
| --- | --- | --- | --- | --- | --- | --- | --- | --- | --- | --- | --- | --- | --- | --- | --- | --- | --- | --- | --- | --- | --- | --- | --- |
| Fasting blood glucose | PDCA | PHC | PBTC | LNC | PBTC | Tumor size | PDCA | PHC | PBTC | LNR | PDCA | PHC | PBTC | LNM | PDCA | PHC | BMI | PDCA | PBTC | Intraoperative blood loss | PHC | Age | PBTC |
| 0 | 0 | 0 | 0 | 0 | 0 | 1 | 0 | 0 | 0 | 0 | 0 | 0 | 0 | 0 | 0 | 0 | 14 | 38 | 69 | 0 | 0 | 30 | 0 |
| 5 | 7 | 7 | 7 | 5 | 8 | 2 | 5 | 3 | 5 | 0.2 | 3 | 5 | 4 | 2 | 4 | 3 | 16 | 34 | 63 | 500 | 3 | 35 | 4 |
| 10 | 14 | 14 | 14 | 10 | 16 | 3 | 9 | 6 | 10 | 0.4 | 6 | 10 | 8 | 4 | 8 | 7 | 18 | 31 | 57 | 1000 | 5 | 40 | 8 |
| 15 | 21 | 21 | 21 | 15 | 23 | 4 | 14 | 9 | 15 | 0.6 | 10 | 14 | 13 | 6 | 13 | 10 | 20 | 27 | 51 | 1500 | 8 | 45 | 12 |
| 20 | 29 | 29 | 29 | 20 | 31 | 5 | 18 | 12 | 20 | 0.8 | 13 | 19 | 17 | 8 | 17 | 14 | 22 | 24 | 44 | 2000 | 10 | 50 | 16 |
| 25 | 36 | 36 | 36 | 25 | 39 | 6 | 23 | 15 | 24 | 1.0 | 16 | 24 | 21 | 10 | 21 | 17 | 24 | 20 | 38 | 2500 | 13 | 55 | 20 |
| 30 | 43 | 43 | 43 | 30 | 47 | 7 | 27 | 18 | 29 | 1.2 | 19 | 29 | 25 | 12 | 25 | 21 | 26 | 17 | 32 | 3000 | 15 | 60 | 25 |
| 35 | 50 | 50 | 50 | 35 | 55 | 8 | 32 | 22 | 34 | 1.4 | 23 | 33 | 30 | 14 | 29 | 24 | 28 | 14 | 25 | 3500 | 18 | 65 | 29 |
| 40 | 57 | 57 | 57 |  |  | 9 | 36 | 25 | 39 | 1.6 | 26 | 38 | 34 | 16 | 33 | 28 | 30 | 10 | 19 | 4000 | 20 | 70 | 33 |
| 45 | 64 | 64 | 64 |  |  | 10 | 41 | 28 | 44 | 1.8 | 29 | 43 | 38 | 18 | 38 | 31 | 32 | 7 | 13 |  |  | 75 | 37 |
| 50 | 71 | 71 | 71 |  |  | 11 | 45 | 31 | 49 | 2.0 | 32 | 48 | 42 | 20 | 42 | 35 | 34 | 3 | 6 |  |  | 80 | 41 |
| 55 | 79 | 79 | 79 |  |  | 12 | 50 | 34 | 54 |  |  |  |  | 22 | 46 | 38 | 36 | 0 | 0 |  |  | 85 | 45 |
| 60 | 86 | 86 | 86 |  |  | 13 | 54 | 37 | 59 |  |  |  |  | 24 | 50 | 42 |  |  |  |  |  |  |  |
| 65 | 93 | 93 | 93 |  |  | 14 | 59 | 40 | 63 |  |  |  |  |  |  |  |  |  |  |  |  |  |  |
| 70 | 100 | 100 | 100 |  |  | 15 | 63 | 43 | 68 |  |  |  |  |  |  |  |  |  |  |  |  |  |  |

Note: PDAC, pancreatic ductal adenocarcinoma; PHC, pancreatic head/uncinate ductal adenocarcinoma; PBTC, pancreatic body/tail ductal adenocarcinoma; BMI, body mass index; LNC, lymph node count; LNM, lymph node metastasis; LNR, lymph node ratio.

**Suppl. Table 7 Relative hazard for Nomo stages in derivation and external validation cohorts**

|  | PDAC | | | | PHC | | | | PBTC | | | |
| --- | --- | --- | --- | --- | --- | --- | --- | --- | --- | --- | --- | --- |
| Nomo stages | Cut-point | HR | 95% CI | P | Cut-point | HR | 95% CI | P | Cut-point | HR | 95% CI | P |
| Training cohort |  |  |  |  |  |  |  |  |  |  |  |  |
| nomo1 | <77.60 | ref |  |  | <31.86 | ref |  |  | <120.66 | ref |  |  |
| nomo2 | <86.72 | 2.20 | 1.38-3.49 | 0.0008 | <38.71 | 2.28 | 1.18-4.39 | 0.01 | <131.90 | 2.03 | 1.07-3.87 | 0.03 |
| nomo3 | <92.42 | 2.21 | 1.38-3.52 | 0.0010 | <42.51 | 2.57 | 1.37-4.83 | 0.003 | <142.12 | 1.70 | 0.89-3.26 | 0.104 |
| nomo4 | <98.00 | 2.88 | 1.82-4.57 | <0.0001 | <47.29 | 2.68 | 1.41-5.08 | 0.002 | <149.45 | 2.13 | 1.14-3.99 | 0.01 |
| nomo5 | <103.99 | 3.43 | 2.19-5.39 | <0.0001 | <50.93 | 3.07 | 1.64-5.75 | 0.0004 | <159.23 | 3.14 | 1.68-5.87 | 0.0003 |
| nomo6 | <109.53 | 2.86 | 1.81-4.51 | <0.0001 | <54.76 | 3.11 | 1.63-5.94 | 0.0005 | <165.94 | 3.63 | 1.92-6.85 | <0.0001 |
| nomo7 | <116.96 | 5.42 | 3.47-8.46 | <0.0001 | <59.75 | 4.16 | 2.28-7.58 | <0.0001 | <175.63 | 4.38 | 2.36-8.14 | <0.0001 |
| nomo8 | <125.07 | 4.85 | 3.12-7.56 | <0.0001 | <65.73 | 5.45 | 2.99-9.94 | <0.0001 | <184.64 | 6.97 | 3.72-13.06 | <0.0001 |
| nomo9 | <138.93 | 7.69 | 4.95-11.94 | <0.0001 | <76.51 | 6.20 | 3.39-11.31 | <0.0001 | <199.67 | 8.05 | 4.33-14.99 | <0.0001 |
| nomo10 | 138.93+ | 10.99 | 7.08-17.05 | <0.0001 | 76.51+ | 11.33 | 6.23-20.61 | <0.0001 | 199.67+ | 10.96 | 5.88-20.40 | <0.0001 |
| Test cohort |  |  |  |  |  |  |  |  |  |  |  |  |
| nomo1 | <81.83 | ref |  |  | <34.21 | ref |  |  | <123.67 | ref |  |  |
| nomo2 | <89.91 | 1.33 | 0.70-2.52 | 0.38 | <39.97 | 2.28 | 0.89-5.81 | 0.08 | <136.12 | 0.83 | 0.31-2.25 | 0.72 |
| nomo3 | <95.98 | 1.62 | 0.87-3.03 | 0.12 | <46.21 | 3.67 | 1.47-9.15 | 0.005 | <147.17 | 2.83 | 1.21-6.61 | 0.016 |
| nomo4 | <101.07 | 1.85 | 1.00-3.44 | 0.04 | <49.99 | 2.61 | 1.02-6.66 | 0.04 | <152.24 | 2.19 | 0.91-5.24 | 0.076 |
| nomo5 | <106.42 | 2.23 | 1.19-4.19 | 0.01 | <53.89 | 4.49 | 1.82-11.07 | 0.001 | <160.46 | 3.03 | 1.28-7.13 | 0.011 |
| nomo6 | <111.76 | 2.00 | 1.06-3.75 | 0.03 | <57.21 | 3.02 | 1.21-7.51 | 0.01 | <168.47 | 2.70 | 1.11-6.55 | 0.027 |
| nomo7 | <119.17 | 2.67 | 1.46-4.88 | 0.001 | <61.43 | 3.02 | 1.18-7.69 | 0.02 | <176.78 | 2.33 | 0.98-5.56 | 0.054 |
| nomo8 | <129.78 | 4.01 | 2.20-7.34 | <0.0001 | <66.01 | 3.34 | 1.34-8.30 | 0.009 | <187.13 | 4.32 | 1.79-10.39 | 0.001 |
| nomo9 | <139.22 | 5.00 | 2.75-9.07 | <0.0001 | <76.51 | 4.37 | 1.79-10.67 | 0.001 | <201.56 | 5.12 | 2.18-12.03 | 0.0001 |
| nomo10 | 139.22+ | 6.42 | 3.56-11.59 | <0.0001 | 76.51+ | 8.72 | 3.64-20.90 | <0.0001 | 201.56+ | 6.61 | 2.72-16.04 | <0.0001 |

Note: PDAC, pancreatic ductal adenocarcinoma; PHC, pancreatic head/uncinate ductal adenocarcinoma; PBTC, pancreatic body/tail ductal adenocarcinoma.
